# Wnt5a is a TLR2/4-ligand that induces tolerance in human myeloid cells

**DOI:** 10.1038/s42003-019-0432-4

**Published:** 2019-05-09

**Authors:** Meliha Mehmeti, Caroline Bergenfelz, Eva Källberg, Camilla Rydberg Millrud, Per Björk, Fredrik Ivars, Bengt Johansson-Lindbom, Sven Kjellström, Ingemar André, Karin Leandersson

**Affiliations:** 1 Cancer Immunology, Department of Translational Medicine, Lund University, Malmö, 21428 Sweden; 2 Experimental Infection Medicine, Department of Translational Medicine, Lund University, Malmö, 21428 Sweden; 3 Immunology Unit, Department of Experimental Medicine, Lund University, Lund, 22184 Sweden; 4 Active Biotech AB, Lund, 22007 Sweden; 5 Centre of Excellence in Biological and Medical Mass Spectrometry, Lund University, Lund, 22184 Sweden; 6 Center for Molecular Protein Science, Lund University, Lund, 22362 Sweden

**Keywords:** Immune evasion, Monocytes and macrophages

## Abstract

Innate immune responses are rapid, dynamic and highly regulated to avoid overt reactions. This regulation is executed by innate immune tolerance mechanisms that remain obscure. Wnt5a is a signalling protein mainly involved in developmental processes and cancer. The effect of Wnt5a on inflammatory myeloid cells is controversial. Here, we combine primary cell cultures, in vitro binding studies, mass spectrometry and *Drosophila* protein modelling to show that Wnt5a is a direct ligand of toll-like receptor (TLR) 2 and 4. The binding promotes a MyD88-non-canonical nuclear factor of kappa B (NFκB) and AP-1 signalling cascade, with contradictory profiles in mouse (pro-inflammatory) and human (anti-inflammatory) myeloid immune cells. These data reveal that the true nature of Wnt5a in inflammatory cells, is to regulate TLR signals, and in human myeloid cells it acts as an endogenous, tolerance-associated molecular pattern (TAMP), inducing IL-10 and innate immune tolerance.

## Abstract (web summary)

Meliha Mehmeti et al. show that Wnt5a is a direct ligand of toll-like receptor 2 and 4 to act as an endogenous tolerance-associated molecular pattern, triggering innate immune tolerance in human myeloid cells. This study expands the functional repertoire of Wnt5a that is mainly known as a signalling protein involved in development and cancer.

## Introduction

The immune system is divided into an innate immune response that reacts immediately with specificity towards exogenous and endogenous dangers, and an acquired immune response, that reacts with a delay but with high specificity eventually leading to immunity. Both sides of the immune system are tightly regulated by a mechanism called immunological tolerance that is necessary to avoid harmful overt reactions. Immunological tolerance has an important role in many diseases. This is relevant in the case of acute life-threatening inflammation that can be caused by severe infections and auto-immune disorders, and also in cancer, as immune tolerance is hijacked by the tumour to avoid destruction by the immune cells^1^.

Immunological tolerance is an important mechanism regulating a potentially harmful, overt immune response in various diseases. While tolerance of the adaptive immune system has been thoroughly investigated, that of the innate immune system is less understood. It is therefore of interest to clarify the molecular mechanisms behind the innate immune cell tolerance, and that of monocyte tolerance in particular, especially since this mechanism is responsible for potentially life-threatening conditions, such as immune paralysis in sepsis and immunosuppression in cancer^1–3^. For instance, in patients with sepsis, Wnt5a and other Wnt-family proteins are strongly upregulated in the serum and bone marrow^4,5^. The physiological role of this upregulation, at sites with high content of inflammatory cells, has remained unexplored.

Myeloid cells are innate immune cells that can either be pro- or anti-inflammatory, depending on the surrounding microenvironment and signalling network^2^. Crucial activating receptors for myeloid cells are Toll-like receptors (TLRs) that are part of the pattern recognition receptor family^6^. The downstream signal induced by these receptors promotes rapid induction of pro- and anti-inflammatory mediators with broad functions to orchestrate the ensuing immune response. The pro-inflammatory mediators are important to fight pathogens, induce Th1 and Th17 responses and clear wounds upon damage, and the anti-inflammatory that is important to promote wound healing mechanisms, Th2 responses and most importantly to terminate and regulate the initial pro-inflammatory immune reactions by inducing innate immune tolerance. The role for TLRs in tolerance induction is still unclear^7^.

The plasticity paradigm of innate myeloid cells during an immune reaction is best represented by the time course of sepsis. The onset of sepsis is characterized by a systemic inflammatory response syndrome, which is subsequently counteracted by a compensatory anti-inflammatory response syndrome that induces systemic tolerance and immune paralysis^3,7,8^. The syndrome is currently explained by the widely accepted endotoxin tolerance theory^7,9^. According to that theory, a low dose of a ligand of a TLR [e.g. lipopolysaccharide (LPS)] and certain cytokines [e.g. interleukin (IL) 10] render myeloid cells incapable of responding to a secondary TLR-stimulation. The exact molecular mechanism is as yet undefined, but downregulation of TLRs and formation of NFκB p50-homodimers that promote IL-10 transcription, while inhibiting TNFα, has been suggested^7,10–12^. One endogenous mediator that has been implicated to drive the molecular mechanism of innate tolerance is Wnt5a^13–15^.

Wnt5a is a non-canonical Wnt-protein involved in developmental processes, inflammation, and certain forms of cancer. The signalling receptors of Wnt5a described to date are the transmembrane receptor families Frizzled, ROR2, and Ryk. Wnt5a binding to the Frizzled receptors affects cellular polarity, migration, and adhesion^16,17^. Wnt5a signals locally in auto- and paracrine manners, utilizing heparin sulfate proteoglycans at the cell surface^18^. The role and signalling cascade for Wnt5a in inflammatory cells has long been debated^4,13–15,19–26^. We have previously shown that Wnt5a affects cytokine production in myeloid and tumour cells^13,19,27^. In human myeloid cells, Wnt5a mainly induces the production of the anti-inflammatory mediator IL-10. This leads to the induction of tolerance in human monocytes and macrophages^13,19^. Similar IL-10–mediated effects by Wnt5a were also described in mouse^14^ and human dendritic cells^15^. However, Wnt5a was also suggested to act as a pro-inflammatory factor in mouse^4,20^, with the molecular effect suggested to be dependent on the Wnt5a receptors Frizzled-5, Frizzled-1, or ROR2^4,20,22^. In *D. melanogaster*, according to early studies on the non-canonical Wnt-protein WntD, WntD is crucial for the inhibition of Toll/TLR-induced inflammatory conditions^28^. In the current study, we explored the link between Wnt5a and TLR in innate immune cell tolerance.

Here we demonstrate that Wnt5a, a Wnt-protein, is a TLR ligand and a tolerance-associated molecule, emphasizing its role under inflammatory conditions. We show that the Wnt5a-induced signalling generates different outcomes in human and mouse, with anti-inflammatory mediators and effector cells induced in human and pro-inflammatory mediators induced in mouse. We also show that Wnt5a itself is upregulated by TLR-stimuli, similarly to what has been described in *D. melanogaster*^28^. This reflects a true homeostatic feedback loop between Wnt5a and TLR (Toll in *D. melanogaster*) in inflammatory cells of human and fly origin, in which Wnt5a regulates the level of Toll/TLR signalling. We propose that Wnt5a is an endogenous, tolerance-associated molecular pattern (TAMP) in humans, and to our knowledge, the first Wnt-protein shown to bind a pattern recognition receptor, thus revealing its crucial role in inflammatory conditions.

## Results

### Wnt5a expression is induced by endotoxin in primary human myeloid cells

**Fig. 1:**
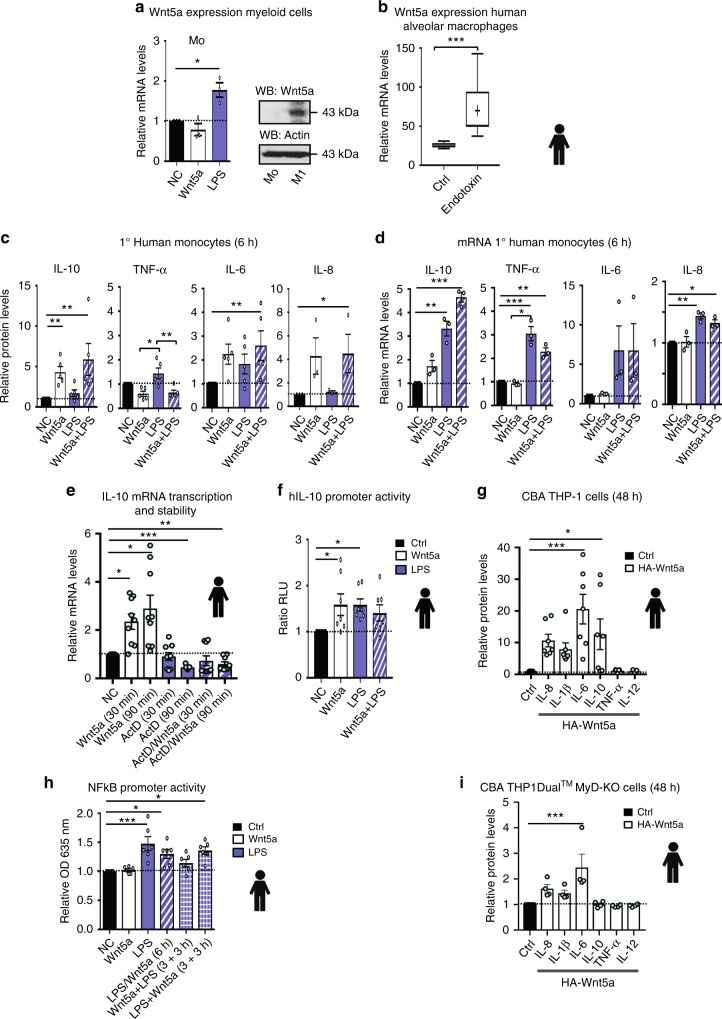
Wnt5a expression is induced by endotoxin and promotes anti-inflammatory IL-10 in primary human myeloid cells. **a** Wnt5a expression in primary human monocytes (left, mRNA as assessed by RT-qPCR) and primary human monocyte derived M1 macrophages (right, protein as assessed by western blotting). Wnt5a or LPS (24 h) in the absence of serum (serum free; SF). (*n* = 3) Dunnett’s test was used for multiple comparisons. **b** LPS induces Wnt5a expression in human primary human alveolar macrophages, as evidenced by analysis of published GEO datasets. Mann–Whitney *t*-test. **c** Wnt5a induces anti-inflammatory cytokines in primary human monocytes. Primary human monocytes from healthy controls were treated with rWnt5a and/or LPS (6 h SF). The levels of secreted IL-10, TNFα, and IL-6 were analysed by CBA (*n* = 5); IL-8 by ELISA (*n* = 3). ANOVA (no brackets) or Student’s *t*-test (brackets). For multiple comparisons Dunn’s test was used. **d** Wnt5a induces transcription of *IL10* mRNA in primary human monocytes. (*n* = 3) Dunnett’s test was used for multiple comparisons. **e** Wnt5a does not enhance *IL10* mRNA stability in primary human monocytes. Actinomycin D (ActD). The mRNA levels were determined using RT-qPCR. (*n* = 9) Dunnett’s test was used for multiple comparisons. **f** Wnt5a induces activation of the *IL10* promoter. Luciferase IL-10 promoter assay was performed using human THP-1 cells transfected with HA-Wnt5a or stimulated with LPS (18 h). Relative dual luciferase units (RLU) were determined, with the pRL-TK vector as the control. (*n* = 7) Dunnett’s test was used for multiple comparisons. **g** Overexpression of pcDNA3-HA-Wnt5a (48 h) in THP-1 cells induces cytokine expression. Cytokines were analysed by CBA. (*n* = 7) Dunnett’s test was used for multiple comparisons. **h** Wnt5a does not induce NFκB activity. THP-1- Blue™ NFκB cells were treated with rWnt5a and/or LPS (6 h). (*n* = 6). Dunnett’s test was used for multiple comparisons. **i** Overexpression of pcDNA3-HA-Wnt5a (48 h) in THP1-Dual^TM^ MyD-KO cells reduces cytokine expression as compared to **g**. Cytokines were analysed by CBA. (*n* = 4) Dunnett’s test was used for multiple comparisons. Control cells were transfected with an anti-sense pcDNA3-as-Wnt5a vector. Error bars indicate SEM; *p < 0.05, **p < 0.01, and ***p < 0.001. Data were analysed by ANOVA or *t*-test as indicated

Regulation of *Wnt5a* expression in mammalian cells is obscure. One of the few signals known to directly upregulate *Wnt5a* expression is endotoxin (LPS). LPS induced *Wnt5a* both in vitro, in primary human monocytes (Fig. 1a, left) and macrophages (Fig. 1a, right), and in vivo, in primary human alveolar macrophages, as determined by analysing publicly available gene expression array dataset^29^ from NCBI Gene Expression Omnibus profiles^29,30^ (Fig. 1b). Addition of Chloroquine (CQ) to inhibit endosomal TLR-signalling did not affect the LPS-induced expression of *Wnt5a* in primary human monocytes (Supplementary Fig. 1a), indicating that conventional MyD88-dependent TLR-signals were responsible.

### Wnt5a induces anti-inflammatory cytokines and tolerance in human myeloid cells

We have previously shown that stimulation of primary human monocytes with rWnt5a in combination with typical TLR4 ligands promotes the differentiation of immunosuppressive monocytic—myeloid derived suppressor cells (Mo-MDSC) or M2 macrophages from primary human monocytes in vitro^13^. We showed that this effect was exerted by the non-canonical Wnt5a signal on the NFκB intracellular cascade, promoting p50-homodimer formation that induced IL-10 production, the mechanism underlying innate immune cell tolerance^13^. This effect was observed with both pathogen-associated molecular patterns (PAMP: LPS) and damage-associated molecular patterns (DAMPs: HMGB1 or S100A9^31^) (Supplementary Fig. 1b–d).

To investigate the above findings in more detail, we analysed the expression pattern of cytokines produced upon a short (6-h) Wnt5a stimulation of primary human monocytes in vitro. Wnt5a stimulation alone induced IL-10, IL-6, and IL-8 protein levels, while reducing TNFα levels (Fig. 1c). Neither IL-1β, PGE2 nor IL-12 was produced after this short stimulation (Supplementary Fig. 2a). A similar effect was observed when Wnt5a was used in combination with LPS; in fact, Wnt5a reduced the LPS-induced TNFα release (Fig.1c and Supplementary Fig. 2d). The Wnt5a-induced response was rapid and declined after 24 h, in contrast to the sustained effect of LPS or LPS combined with Wnt5a (Supplementary Fig. 2b, c). Further, rWnt5a was more effective in the absence of serum in vitro, while the stimulatory capacity of LPS alone was low under these conditions, most likely because of the absence of serum proteins, as previously described^32^ (Fig. 1c and Supplementary Fig. 2a–d). Hence, serum supplementation was used as positive control for LPS stimulation (Supplementary Fig. 2d).

### Wnt5a induces IL10 transcription

Changes in the *IL10* and *TNFA* mRNA levels in Wnt5a-treated primary human monocytes corresponded to protein levels (Fig. 1c, d). As shown, *IL10* mRNA levels increased (Fig. 1d, e) and *TNFA* mRNA levels decreased (Fig. 1d), although Wnt5a-induced inhibition of LPS-induced *TNFA* mRNA levels was less apparent than on protein level (Fig. 1c, d). The Wnt5a-induced *IL10* expression was rapid, and clearly pronounced already after 30- and 90-min induction (Fig. 1e). Indeed, Wnt5a affected the *IL10* transcription per se by inducing *IL10* mRNA levels (Fig. 1d, e) and *IL10* promoter activity (Fig. 1f), but not mRNA stability (Fig. 1e), as indicated by the decay of *IL10* mRNA upon treatment with the transcriptional inhibitor actinomycin D (ActD) and rWnt5a. For IL-6 and IL-8, only the protein but not the mRNA levels were affected by Wnt5a stimulation, in line with previous findings^27^ (Fig. 1c, d).

The above observations were not unexpected, as stimulation of cytokine expression in myeloid cells by rWnt5a has been previously shown^24^. However, the previous findings led to speculation that the used rWnt5a preparation may have been LPS-contaminated^24^, despite various tests disproving such claims^13^. Hence, the putative endotoxin contamination of the rWnt5a (and rWnt3a) preparations used in the current study was evaluated using a *Limulus* assay. The assay revealed that the proteins were free of LPS (Supplementary Fig. 2e). We also confirmed the content of rWnt5a by MS analysis (Supplementary Table 2). To avoid the use of recombinant proteins, we next overexpressed human HA-Wnt5a in the human THP-1 monocytoid cell line (Fig. 1g). We have previously shown that ectopic expression of Wnt5a leads to secretion^33^. Accordingly, HA-Wnt5a expression exerted similar effect on cytokine expression as rWnt5a, except that IL-1β levels were also induced, and at this time point (48 h) TNFα levels were slightly higher than in rWnt5a-stimulated primary human monocytes, probably as a secondary effect (Fig. 1g). Hence, the protein Wnt5a indeed affects the inflammatory protein production in myeloid cells.

Together, these findings indicate that Wnt5a is able to rapidly induce or inhibit specific inflammatory cytokines in primary human monocytes, and to promote an anti-inflammatory phenotype (i.e. tolerance phenotype; Mo-MDSC or M2-like phenotype) in human myeloid cells.

### Wnt5a signalling does not induce classical NFκB-dependent transcription

Because both pro- and anti-inflammatory cytokines are regulated by NFκB transcription factors^34^, we next investigated whether Wnt5a could induce classical NFκB-dependent transcription. We used the NFκB-Blue reporter THP1 cell assay, a sensitive assay in which SEAP levels produced by the cells are monitored by spectrophotometer and correspond to NFκB activity. As shown in Fig. 1h and Supplementary Fig. 2f, Wnt5a stimulation (6 h, 12 h, and 24 h) did not induce classical NFκB-Blue reporter activity, in contrast to both LPS and the DAMP S100A9 (Fig. 1h and Supplementary Fig. 2f, g). Interestingly, and as previously reported^13^, Wnt5a slightly inhibited the LPS-induced NFκB activity (Fig. 1h). The inhibition of LPS-induced NFκB activity by Wnt5a was most evident when the cells were exposed to Wnt5a before LPS (Fig. 1h). Typical LPS-induced activation of NFκB-associated signalling proteins were also analysed, with or without prior HA-Wnt5a transfection of THP-1 cells (Supplementary Fig. 3A). Primary human monocytes stimulated with rWnt5a only were also analysed (Supplementary Fig. 3B). Of the proteins investigated, the kinetics of p-p38, p-ERK1/2 and p-Akt were enhanced by the addition of Wnt5a while degradation of IκBα was decreased. In summary, this indicated that Wnt5a could rapidly regulate the levels of several pro- and anti-inflammatory proteins, despite lacking the classical NFκB-inducing activity.

### Wnt5a/TLR4 induces pro-inflammatory cytokines in mouse myeloid cells

We have been struck by how often conflicting results are reported concerning Wnt5a-induced cytokines in myeloid cells. To investigate whether the Wnt5a-associated induction of innate immune response genes was affected by the classical MyD88-dependent NFκB-signalling pathway, we analysed the responses of Wnt5a in THP1-Dual^TM^ MyD-KO (MyD88 KO; Fig. 1i), and found that the Wnt5a-induced production of IL-10 was completely inhibited, while Wnt5a-induced IL1β, IL-8, and IL-6 were reduced and TNFα not affected, as compared to THP-1 cells (Fig. 1g).

**Fig. 2:**
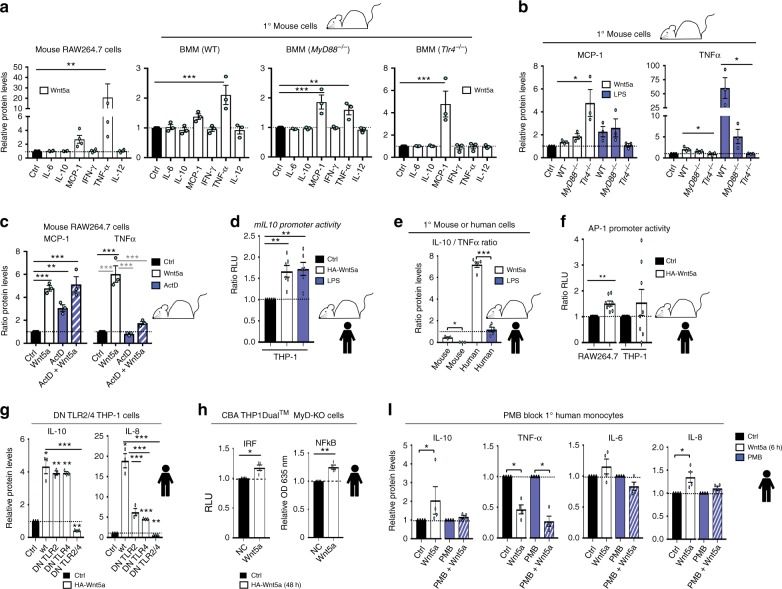
Wnt5a induces the production of pro-inflammatory cytokines in mouse myeloid cells. **a** Murine RAW264.7 macrophages, or primary (1°) WT C57Bl/6, *MyD88*^*-/-*^, or *Tlr4*^*-/-*^ BMM, were treated with rWnt5a (6 h). Cytokine levels were determined by CBA. (*n* = 4 and *n* = 3). Dunnett’s test was used for multiple comparisons. **b** Wnt5a signalling in WT C57Bl/6, *MyD88*^−/−^, and *Tlr4*^−/−^ mice. Relative protein levels of MCP-1 (CCL2) or TNFα from the experiment shown (**a**) in different mouse strains are shown. (*n* = 3). Dunnett’s test was used for multiple comparisons. **c** Wnt5a induces *Tnf*, but not *Ccl2* (MCP-1) mRNA transcription as shown by using the transcriptional blocker actinomycin D (ActD) and RAW264.7 cells. The protein levels were determined using CBA. (*n* = 3) Dunnett’s test (black annotation) and Holm-Sidak´s test (grey annotations) was used for multiple comparisons. **d** HA-Wnt5a-induced activation of the mouse *Il10* promoter in human THP-1 cells. LPS (18 h) as positive control. (*n* = 6). Dunnett’s test was used for multiple comparisons. **e** Wnt5a-induced IL-10:TNFα protein level ratio in mouse and human cells. (*n* = 3 and *n* = 5). Data were analysed by paired *t*-test. **f** Wnt5a-induced activation of an AP-1 reporter in mouse RAW264.7 but inconsistently in human THP-1 cells. (*n* = 8) Data were analysed by paired *t*-test. **g** Wnt5a signalling in human myeloid cells is inhibited by ectopic expression of dominant negative (DN)-hTLR2 and DN-hTLR4. DN-TLR2, DN-TLR4 or DN-TLR2/DN-TLR4 plasmids, using THP-1 cells and HA-Wnt5a or Ctrl vector. The protein levels were determined using CBA. The ratio rWnt5a-treated/Ctrl-treated for each pair is shown. (*n* = 3) Holm-Sidak´s test was used for multiple comparisons. **h** Wnt5a slightly affects MyD88-independent IRF and NFκB signalling. Overexpression of pcDNA3-HA-Wnt5a (48 h) in THP1-Dual^TM^ MyD-KO cells. (*n* = 4) Data were analysed by paired *t*-test. **i** Wnt5a signalling in human primary monocytes is blocked by PMB. Relative levels of IL-10, TNFα, IL-6 (CBA), and IL-8 (ELISA) stimulated with rWnt5a (6 h), with or without PMB treatment, are shown. Error bars indicate SEM (*n* = 4). Mann–Whitney U test was used for each bar comparison to Ctrl. Error bars indicate SEM; *p < 0.05, **p < 0.01, and ***p < 0.001. Data were analysed by ANOVA or *t*-test as indicated for each panel

The responses to Wnt5a of primary (1°) bone marrow derived macrophages (BMM) isolated from WT, *MyD88*^−/−^, and *Tlr4*^−/−^ mice were next analysed. In sharp contrast to cells of human origin, stimulation of mouse RAW264.7 macrophages or primary (1°) mouse BMM from WT mice (Fig. 2a) with Wnt5a, induced the pro-inflammatory cytokine TNFα and, to some extent, MCP-1 (CCL2). The same effect, although more pronounced, was observed upon induction with LPS (Supplementary Fig. 4a). Wnt5a did not induce IL-10 production and did not suppress TNFα levels in murine cells under any conditions tested (Fig. 2a). The Wnt5a-induced TNFα production was furthermore entirely TLR4-dependent and partially MyD88 dependent, while the production of MCP-1 was TLR4- and MyD88 independent (Fig. 2a, b). The Wnt5a-induced MCP-1 production was, however, inhibited by addition of Dishevelled (Dvl) inhibitors (Supplementary Fig. 4b), indicating that the Wnt-Frizzled-signalling pathways were responsible for the MCP-1 induction, in sharp contrast to the mouse TNFα induction (Supplementary Fig. 4b). The molecular mechanism behind the Wnt5a-induced TNFα in mouse was proven to be at the transcriptional levels, since the addition of the transcriptional blocker ActD inhibited its induction, while the Wnt5a-induced MCP-1 was not (Fig. 2c).

The fact that IL-10 was not induced by Wnt5a in mouse cells led us to investigate the mouse *Il10* (m*ILl0*) promoter in human cells and found that indeed the mouse *Il10* promoter was activated by Wnt5a in human cells, indicating a difference in the Wnt5a-TLR4-induced signalling cascade in mouse and human myeloid cells (Fig. 2d). Consequently, there was a striking difference in the ratio of Wnt5a-induced IL-10 to TNFα levels in mouse and human cells (Fig. 2e). TLR4-MyD88 induced transcriptional regulation of *Tnf* is caused either by classical NFκB or AP-1 motifs. We thus tested whether Wnt5a possibly could induce AP-1 activation in mouse or human cells. Wnt5a induced a robust activation of the AP-1 reporter in mouse cells, and to a variable extent also slightly in human cells (Fig. 2f). This indicated that the biological effect of Wnt5a in myeloid cells is vastly different in mouse and human, and resolves the reported controversies on the function of Wnt5a in mouse and human myeloid cells^4,13–15,19–24^.

### Wnt5a signals via TLR2/4 pathways in human myeloid cells

The Wnt5a-induced cytokines in human cells were MyD88-dependent, especially IL-10 and IL-8 as shown both in THP1-Dual^TM^ MyD-KO cells and by using MyD88 inhibitors (Fig. 1i and Supplementary Fig. 5a), we therefore next specifically targeted TLR2 and TLR4 in human cells. By overexpressing Dominant Negative (DN)-TLR2 and -TLR4 in THP-1 cells, thus inhibiting any TLR2 or 4-signals (Fig. 2g), we could show that HA-Wnt5a-induced human IL-10 and IL-8 was reduced (Fig. 2g). We also investigated whether Wnt5a could induce the MyD88-independent pathways TRIF/IRF (Luc) and NFκB (SEAP) in THP1-Dual^TM^ KO-MyD assays (Fig. 2h). As shown, in MyD88-KO cells, Wnt5a only modestly induced the IRF and NFκB MyD88-independent pathways (Fig. 2h). A possible intracellular endosomal Wnt5a/TLR-signal pathway inducing *IL10* was however ruled out using Chloroquine (CQ) in primary human monocytes stimulated with rWnt5a (Supplementary Fig. 1a right). To summarize, in human cells the Wnt5a-induced *IL10* is completely reliant on the TLR2/4-MyD88-dependent non-endosomal pathways (shown in Fig. 1i, Fig. 2g and Supplementary Fig. 1a).

Interestingly, blocking TLR2 or TLR4 with polyclonal antibodies against the LPS-binding site did not affect Wnt5a-mediated induction of IL-10 levels (Supplementary Fig. 5b). By contrast, PMB, a cyclic non-ribosomal polypeptide that blocks the interaction between LPS and TLR4 by binding to the lipid A domain of LPS^35^, inhibited the rapid induction of inflammatory cytokines in 1° human monocytes treated with rWnt5a (6 h), but not the Wnt5a-associated inhibition of TNFα levels (Fig. 2i and Supplementary Fig. 5c). This suggested that Wnt5a signals in a unique TLR4-MyD88-dependent fashion distinct from LPS, probably binding via a lipid moiety similar to lipid A in LPS, in both mouse and human myeloid cells.

### Wnt5a is a ligand of TLR4 and TLR2

**Fig. 3:**
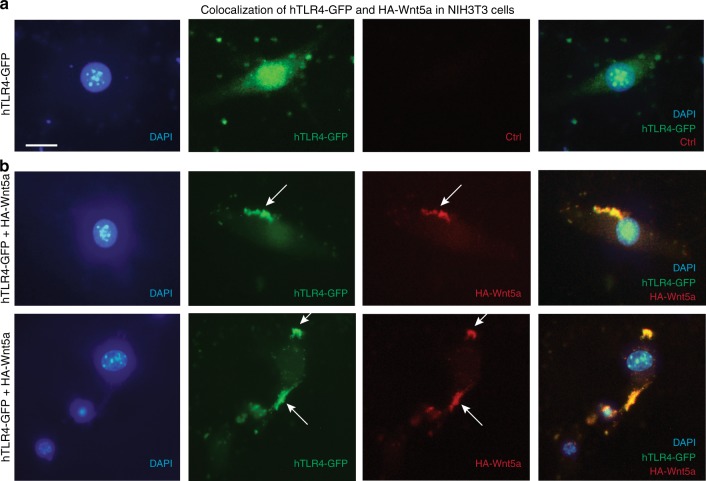
Wnt5a and TLR4 co-localize in NIH3T3 cells. NIH3T3 cells were co-transfected with HA-Wnt5a-pcDNA3 and pUNO-hTLR4-GFP for 48 h. **a** Cells only transfected with pUNO-hTLR4-GFP, TLR4 was dispersed. **b** Cells transfected with both HA-Wnt5a-pcDNA3 and pUNO-hTLR4-GFP, TLR4 and Wnt5a co-localized, forming a cap. HA-Wnt5a was detected using HA-specific Alexa Fluor 594 antibodies; DAPI Vectashield was used for nuclear staining. The images are representative of three replicates. The scale bar represents 10 μm

To explore how Wnt5a might physically affect the TLR receptors, we next investigated the cellular localization of HA-Wnt5a and hTLR4-GFP expressed in NIH3T3 cells. Immunofluorescence with an HA-specific antibody indicated that HA-Wnt5a and hTLR4-GFP colocalized in cells transfected with both protein-encoding constructs (Fig. 3). No TLR-GFP capping was observed in cells transfected only with the hTLR4-GFP–encoding construct (Fig. 3a). This indicated that Wnt5a indeed behaved as a ligand inducing the formation of a TLR4-enriched receptor ligand cluster (Fig. 3b).

**Fig. 4:**
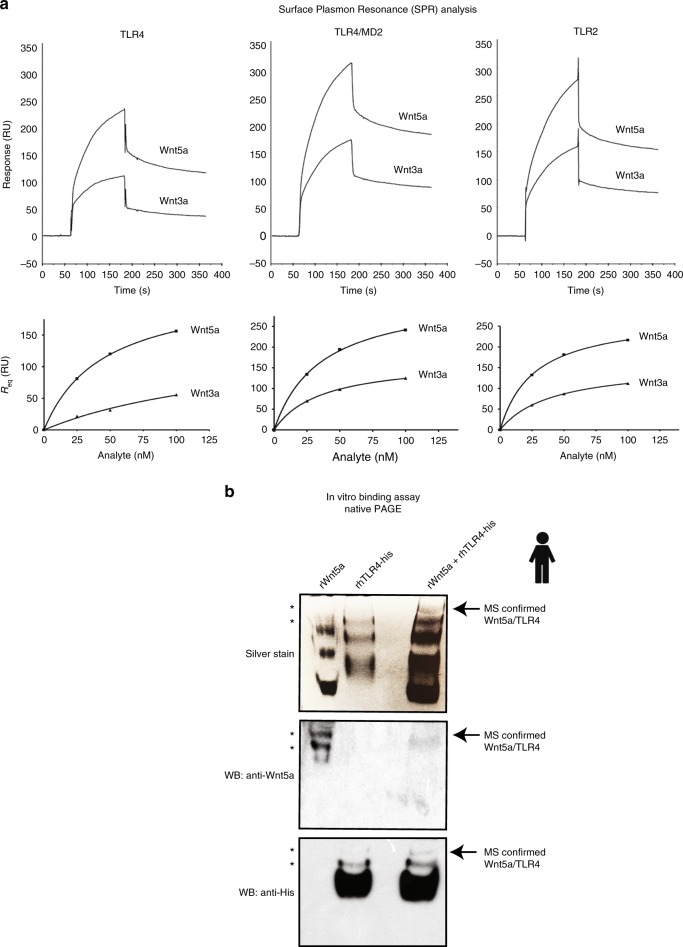
Wnt-proteins interact with TLR proteins. **a** (upper panel) Wnt5a and Wnt3a bind to TLR4, TLR4/MD2, and TLR2. SPR sensorgrams obtained after injection of Wnt protein at 100 nM over TLR4, TLR4/MD2 or TLR2 immobilized in Fc 2, 3, and 4, respectively, are shown. The responses are expressed as resonance units (RU) after subtraction of the sample buffer response in the reference surface (Fc 1). The increase in response at the time of sample injection is a bulk effect caused by traces of CHAPS in the sample solution. (lower panel) Steady-state response (Req), calculated after kinetic evaluation of sensorgrams using a 1:1 model, plotted vs. analyte concentration. The binding curves obtained after injection of Wnt5a and Wnt3a at 25, 50, and 100 nM over TLR4, TLR4/MD2 and TLR2 are shown from left to right in the lower panel. Curves were fit to a one-site hyperbola model for calculation of steady-state affinity and maximal binding. The traces are a representative of two experiments. The results are summarized in Table 1. **b** Wnt5a interacts directly with TLR4. In vitro binding assay for detection of the rWnt5a and rhTLR4-his interaction. The protein band that is only present when both proteins are mixed, and that is specifically recognized by anti-Wnt5a and anti-His antibodies (arrow), contains Wnt5a and TLR4 proteins (as determined by LC-MS/MS; LC-MS/MS data are shown in Supplementary Table 3). Stars, protein bands detected by anti-Wnt5a detects in the well only containing rWnt5a

To further investigate the role of Wnt5a as a possible ligand for TLR2 and TLR4, we performed SPR-based binding assays. Briefly, Wnt5a and Wnt3a as a control (25–100 nM) were injected over TLR4, TLR4/MD2, or TLR2 immobilized on a Biacore sensor chip. As shown in Fig. 4a, sensorgrams obtained with Wnt injected at the highest concentration (100 nM) clearly indicated binding of both Wnt proteins to TLR4 as well as to TLR4/MD2, and TLR2. The data were then used to calculate the kinetic parameters in binding (Fig. 4a, lower panel; Table 1). Wnt5a showed saturable binding to TLR4, TLR4/MD2, and TLR2 with KD values of ~27, 45, and 36 nM, and Bmax values of 274, 226, and 328 RU, respectively. By contrast, although Wnt3a demonstrated affinities in approximately the same range as those for Wnt5a, the Bmax values obtained for Wnt3a were almost two-fold (1.5–1.9 times) lower. Since Wnt3a and Wnt5a are very similar in molecular weight, this could suggest that Wnt5a interacts with TLR as a dimer, but Wnt3a as a monomer. The results are summarized in Table 1.

**Table 1:** Summary of binding data obtained from kinetic analyses^a^

Ligand on surface	Analyte injected	B_max_ (RU)	K_D_ (nM)
TLR4	Wnt5a	226 ± 0.9	44.5 ± 2.0
	Wnt3a	(147 ± 30)^b^	(166 ± 43)^b^
TLR4/MD2	Wnt5a	328 ± 1.8	35.5 ± 5.1
	Wnt3a	170 ± 1.2	36.1 ± 3.0
TLR2	Wnt5a	274 ± 1.5	26.5 ± 4.5
	Wnt3a	158 ± 0.1	41.0 ± 0.2

^a^Binding curves [responses at steady-state (Req) vs. analyte concentration] were fit to a one-site hyperbola model to calculate B_max_ (Response in resonance units (RU)) and K_D_ (± % SE).

^b^Wnt3a showed an almost linear increase in binding to TLR4, explaining the poor fit

Observations from the Biacore analyses were further supported by an in vitro binding assay (Fig. 4b) and LC-MS/MS analysis (Supplementary Table 3), indicating a direct binding between rhTLR4 and rWnt5a.

### Modelling of WntD binding to Toll in Drosophila

**Fig. 5:**
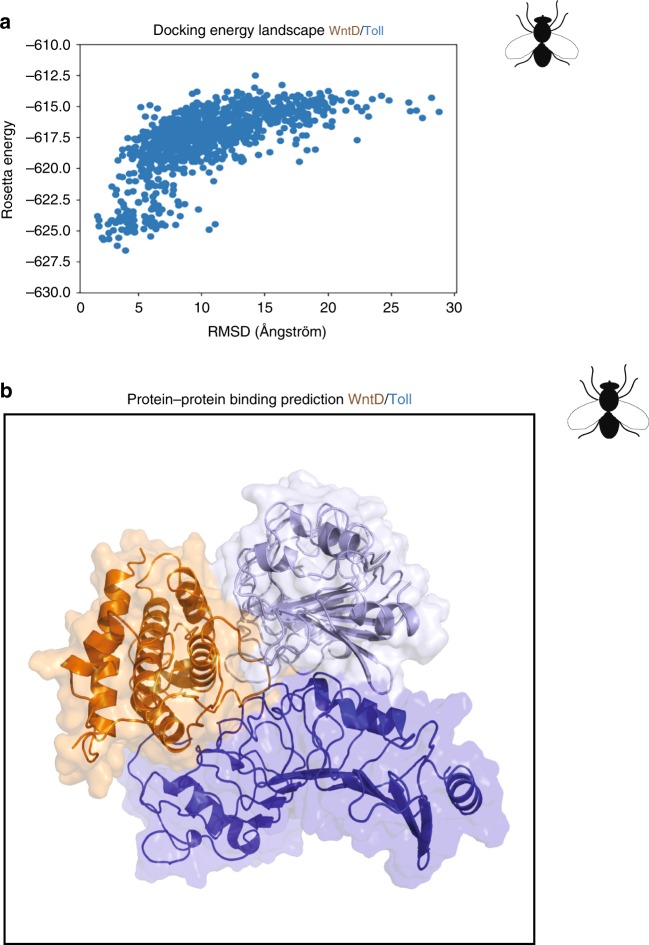
Suggested protein–protein binding model for WntD/Toll. **a** The protein–protein binding between Drosophila Toll (a TLR4 homologue) and WntD (Wnt5a homologue) was modelled using Rosetta macromolecular modelling package^49^. Energy landscapes from the docking perturbation simulations. In this plot the energy of models is plotted against root mean square deviation (rmsd) against the lowest energy model (interface rmsd). **b** A proposed protein–protein docking model of how WntD may interact with Toll

To investigate the structural basis for the interaction between Wnt5a and TLR4 we carried out protein–protein all-atom docking calculations. While the structure of Wnt5a is unknown the Wnt5a *Drosophila* homologue WntD, with similar TLR-NFκB–regulating activity as those presented in the current study^28^, has been crystallized^36^. Global all-atom docking simulations of WntD to a Toll receptor modeled from the crystal structure of Toll was carried out using RosettaDock^36^. The lowest energy model (Fig. 5a) binds at the interface between the two Toll receptor molecule and primarily involves contacts between loop segments on WntD and the Toll receptor solenoid, resulting in a complex with shape complementarity (Fig. 5b). The docking landscape shows a funnel towards the predicted structure, which is typically found for native complexes^36^. While further experimental verification is required, the WntD and Toll model suggest how the homologous Wnt5a may interact with TLR4 and provide a basis for guiding experiments to identify residues critical for complex formation.

## Discussion

In the current study, we explored the link between Wnt5a and TLR in innate immune cell tolerance. We show that Wnt5a signals via TLR4 and TLR2 receptors and represents a novel ligand of TLR, inducing innate immune tolerance in human, but activation in mouse. Further, we demonstrate that the different cellular responses to Wnt5a in mouse and human are underpinned by a difference in the type of induced cytokines, mainly IL-10 and TNFα, in cells of these origins.

**Fig. 6:**
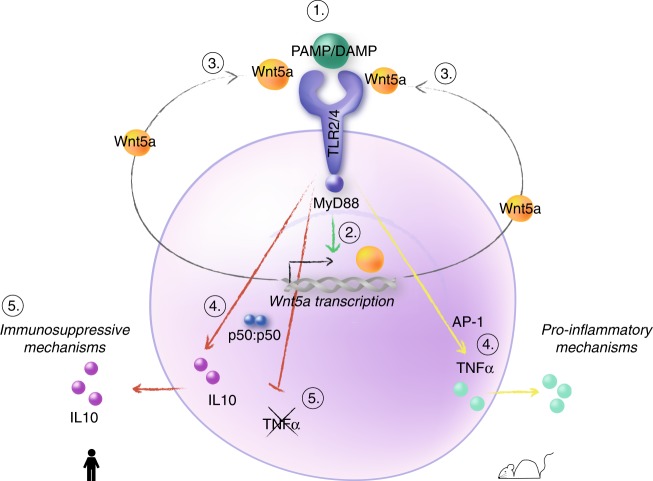
Model for Wnt5a as a tolerance-associated molecular pattern (TAMP). The proposed model for the homeostatic Wnt5a (TAMP)-TLR4 feedback inhibition in human myeloid cells (red arrows) or DAMP signal in mouse (yellow arrow). **1** A danger signal [e.g., endotoxin (PAMP) or endogenous DAMP] binds TLR4 during infection or cell damage. **2** The TLR4-induced signal leads to an increased expression of Wnt5a. **3** Wnt5a is produced and released, and binds TLR2 and TLR4. **4** The Wnt5a/TLR-induced signal promotes TLR-MyD88-NFκB p50-homodimer formation and induction of the immunosuppressive cytokine IL-10 (dominant in human) and TLR-MyD88-MAPK-AP-1 (dominant in mouse) inducing TNFα in mouse. **5** IL-10 promotes the inhibition of TNFα and induction of the anti-inflammatory Mo-MDSC/M2 phenotype in human

Similarly to what we demonstrated for human myeloid cells in the current study, Toll/TLR-signalling induces the expression of WntD in the fly^28^. Further, in infection models of Wnt5a (mouse) or WntD (*D. melanogaster*) deficiency, an attenuated phenotype was observed in the former (because of reduced levels of pro-inflammatory cytokines)^37^, and in sharp contrast, an increased lethality was reported in the latter^28^. This was ascribed to the fact that the non-canonical WntD protein is a feedback inhibitor of Dorsal/NFκB-induced inflammation^28^. Our findings demonstrate that Wnt5a is a key regulator of a similar negative homeostatic feedback mechanism in human. Namely, Wnt5a expression is induced upon endotoxin stimulation in primary human myeloid cells, which is followed by competitive TLR4-binding by Wnt5a, induction of the anti-inflammatory IL-10, and inhibition of TNFα (IL-10 *on* TNFα *off*) (Fig. 6). The proposed mechanism might constitute one of the possible molecular mechanisms underpinning the endotoxin tolerance theory. If such homeostatic feedback mechanism is indeed operational in human myeloid cells during infections, cancer, and inflammation merits further investigation in the clinical setting^4,20,38,39^.

It was somewhat surprising that the molecular mechanism behind the Wnt5a/TLR4-induced tolerance was triggered by, Wnt5a itself, upon specifically binding to the extracellular domains of TLR4 and TLR2, and acting as a novel endogenous ligand of these receptors. Considering the fact that *Drosophila* WntD also inhibits Toll-signalling, and that Wnt5a signals in a TLR4-dependent way also in mouse, we hypothesize that this binding is conserved among species, and therefore provide a suggested protein–protein model using WntD/Toll as model proteins. The binding is positively affected by the presence of MD2, but the downstream signal is severely affected by loss of MyD88. The induction of IL-10 by Wnt5a in human myeloid cells is thus fully dependent on TLR2/4-MyD88-non-endosomal pathways. It is affected by PMB^35,40^, but is not blocked by TLR-blocking antibodies that inhibit the binding of typical endotoxins. Since PMB blocks the interaction between endotoxin and its receptors by binding to the lipid A domain of the former^35^, a similar binding mechanism may be attributed to the neutralization of Wnt5a. A limitation in this study is the lack of crystal structures for any mammalian Wnt5a, thus the modelling of WntD/Toll binding requires further experimental verification for mammalian Wnt5a/TLR in the future.

The observation that Wnt5a does not induce *IL6*, *IL8*, and *Ccl2* transcription indicates that these cytokines might be regulated at the level of Wnt5a-TLR4-PI3K-induced or other Wnt5a-induced cytokine exocytosis^27,41^ rather than by TLR4-MyD88-NFκB or TLR4-MyD88-MAPK-AP-1 induced transcription. The cellular anti-inflammatory effect of the Wn5a-TLR4 interaction in human cells is most likely caused by NFκB p50-homodimer formation as previously shown by us^13^, driving human *IL10* transcription while inhibiting *TNFA*, similarly to the mechanism of endotoxin tolerance of monocytes^7^ (Fig. 5). IL-10 both inhibits TNFα transcription and induces nuclear localization of S100A9, promoting myeloid cell tolerance^7,37^. In contrast, in mouse, Wnt5a-TLR4-induced signalling does not induce IL-10 but TNFα, now clarifying previous contradictory findings concerning Wnt5a in human and mouse and myeloid cells^4,13–15,19–24,37^. Indeed, in mouse, Wnt5a induces a steady AP-1 activity that can promote *Tnf* transcription, rather than *IL10*^42,43^. Of note, it has previously been shown that TNFα induced by TLR4-MyD88-MAPK-AP-1 is not inhibited by IL-10, while TLR4-MyD88-NFκB is^43^. The difference in the nature of the cytokine profiles in mouse and human may be explained both by differences in the Wnt5a-TLR4-MyD88-induced signal in mouse and human as proposed, and by genetic differences in the mouse and human IL-10 promoter^44^. Another explanation could be the TLR profile of the target cell, since TLR2 favours IL-10 production^45^; however, as indicated here, TLR2 and TLR4 seem to be complementary for Wnt5a-induced IL-10 in human cells. Indeed, the source of mouse and human myeloid cells used here differ. Based on the presented findings, we suggest that in human cells and *D. melanogaster*, Wnt5a/WntD should be viewed as a “tolerance-associated molecular pattern” (TAMP) (anti-inflammatory), and in mouse as a damage-associated molecular pattern (DAMP) (pro-inflammatory).

Wnt-proteins have historically typically been shown to bind Frizzled, and ROR2 or Ryk receptors, and the signalling pathways downstream of the non-canonical Wnt signalling affect Ca^2+^/CAMKII and RhoGTPase activity^16^. We now show here that Wnt5a also binds TLRs, and provide a suggested protein–protein binding model using *Drosophila* WntD/Toll as model proteins. How Wnt5a would be able to affect the typical pattern recognition receptor-induced innate immune response genes has until now remained unanswered. Another long-unresolved issue was why in some tissues and under some conditions, the canonical β-catenin–inducing Wnt3a exerted similar, although weaker, effects to Wnt5a. We show here that Wnt3a also binds to TLR4 and TLR2, albeit with a lower affinity than Wnt5a. This explains why we, and others, have seen that the canonical Wnt5a control, Wnt3a, behaves similarly under certain conditions and experiments^13,21,24–26^. Indeed, Frizzled receptors are likely the main receptors for Wnt proteins on somatic cells, but probably not on innate immune cells. Nevertheless, Wnt5a does have TLR-independent and Dvl (Frizzled)-dependent signalling effects also on innate immune cells as exemplified here by mouse CCL2, and binding of Wnt5a to TLRs and simultaneous binding to any of its custom receptors (Frizzled, ROR2, and Ryk), will affect signalling networks that prompts further investigation. This study is limited to using only Wnt5a, Wnt3a, and TLR2, TLR4 as model proteins as our theory all originated from an endotoxin tolerance context^7,13^, and to be able to tell the general importance of this novel interaction one would need to investigate possible additional interactions between Wnt-proteins and pattern recognition receptors in general. Also, the concept of innate immune memory and epigenetic training is an aspect of future interest. We show in the current study that in human myeloid cells, Wnt5a can act as a TAMP, thus regulating rather than inducing activation. The effects of Wnt5a on other TLR4-expressing cells of various origin, such as tumor cells expressing TLR4, should however be investigated. Since many Wnt proteins are induced in myeloid cells during sepsis^5^, the presented findings raise the possibility that Wnt5a and Wnt3a might not be the only TLR ligands from the Wnt family. The notion that they all might be DAMPs or TAMPs with direct functions in the innate immune response is intriguing and warrants further investigation.

## Methods

### Ethics statements

Permission for the study was obtained from the Regional Ethics Committee at Lund University (Dnr (registration number) 2012/689 and Dnr 2014/669) and the participating individuals provided a written informed consent before the study. All mouse procedures were approved by the Malmö/Lund regional ethics committee for animal research (registration number M30-14). *Tlr4*^−/−^ mice (Tacon-IC, Hudson, NY) or *MyD88*^−/−^ mice were bred in the C57Bl/6 background, and maintained at the Department of Microbiology, Immunology, and Glycobiology (*MyD88*^−/−^) or the Biomedical Center (*Tlr4*^−/−^) at Lund University. The study complies with all relevant ethical regulations.

### Human monocyte isolation

Permission for using human blood cells in the study was obtained from the Regional Ethics Committee at Lund University (Dnr (registration number) 2012/689 and Dnr 2014/669) and all the participating individuals provided a written informed consent before the study. Leukocytes from healthy blood donors were isolated from leukocyte concentrates as previously described^13,19^. Briefly, the concentrated leukocytes were diluted in PBS containing 5 mM EDTA and 2.5% w/v sucrose and centrifuged with Ficoll-Paque Plus (Amersham Bioscience, Uppsala, Sweden) to obtain peripheral blood mononuclear cells (PBMC). Monocytes were isolated using monocyte isolation kit II (Miltenyi Biotec, Bergisch Gladbach, Germany) according to the manufacturer’s protocol.

### Mouse macrophage isolation

The tibiae and femurs from WT (C57BL/6), *MyD*88^−/−^, and *Tlr4*^−/−^ mice bred in the C57BL/6 background^25^ were crushed using a mortar and pestle, and the cells were collected in ice-cold PBS. The cells were filtered through a 70-μm cell strainer, counted, and re-suspended in RPMI-1640 medium (Invitrogen, Thermo Fischer Scientific, Waltham, MA, USA) supplemented with 10% heat-inactivated fetal calf serum, 2 mM l-glutamine, 10 mM HEPES (Sigma–Aldrich, St Louis, MO, USA), and 10% (vol/vol) macrophage colony stimulating factor (M-CSF)–containing supernatant from 3T3-CSF cells. Bone marrow cells were seeded on non-tissue culture treated petri dishes (150 x 15 mm) at a density of 10 x 5 cell/ml, and incubated at 37 °C under 5% CO_2_. Upon confluency, the cells were harvested and frozen for later experiments.

### Compounds and cytokine analysis

All recombinant human compounds were purchased from R&D Systems (Minneapolis, MN, USA). The following concentrations were used in cell cultures: 0.5 μg/ml recombinant human/mouse (rh/m)Wnt5a (rWnt5a; molecular weight 38,000; evaluated in a dose-range experiment (Supplementary Fig. 5d)), 0.25 μg/ml rmWnt3a (rWnt3a; molecular weight 37,000), 10 ng/ml rhGM-CSF, 20 ng/ml rhIFN-γ, and 0.1 μg/ml HMGB1. For the in vitro binding assays, rhTLR4 (2.2 μg) and rWnt5a (0.20 μg) were used. LPS from *Salmonella* Typhimurium (0.1 μg/ml), polymyxin B (PMB) (used at 25 μg/ml), and actinomycin D (10 μg/ml) were purchased from Sigma–Aldrich, and used at the indicated concentrations. Chloroquine (CQ) (InvivoGen, Toulouse, France) used at 100 μM. Recombinant human S100A9 (rhS100A9) was a gift from Active Biotech AB (Lund, Sweden). Dishevelled inhibitors NSC (Tocris Bio-Techne, Abington, UK, used at 10 μM) and Dvl (Merck, Darmstadt, Germany, used at 100 μM) were used according to the manufacturers’ protocols. For TLR4 or TLR2 inhibition studies, cells were incubated with pAb-hTLR4 (pab-hstlr4), pAb-hTLR2 (pab-hstlr2), or a pAb-Control (pab-sctr) (Invivogen, Toulouse, France) at a concentration of 0.5 μg/ml for 1 h prior to the stimulation, or a MyD88 inhibitor and control peptide set NBP2-29328 (Novus Biologicals, Littleton, CO, USA) or PMB (Sigma–Aldrich). Supernatants were analysed using cytokine bead array (CBA; BD Biosciences, San Jose, CA, USA) or IL-6, IL-8, IL-10, or PGE2 Quantikine ELISA (R&D Systems) according to the manufacturers’ protocols. Endotoxin levels were determined using a *Limulus* amebocyte lysate assay (GenScript, Piscataway, NJ, USA). Plasmid pcDNA3-HA-Wnt5a was a kind gift from Dr. M. Sen^46^. Plasmids pUNO-GFP-hTLR4, pUNO1-hTLR02-DN, and pUNO1-hTLR04-DN (Invivogen), human IL10 (h*IL10*) promoter plasmid was a kind gift from Professor L. Ziegler-Heitbrock, mouse IL10 (m*Il10*) promoter plasmid^42^ (Addgene, Cambridge, MA, USA), AP-1-reporter kit (BPSBioscience, San Diego, CA, USA), TK-Renilla-promoter (Promega, Madison, WI, USA), anti-HA-Alexa 594 1:2000 (Biolegend, San Diego, CA, USA), Vectashield DAPI (Vectorslab, Burlingame, CA, USA) were used in immunofluorescence experiments. In the native SDS page analyses anti-Wnt5a 1:2000 (clone AF645) from R&D Systems (Minneapolis, MN, USA) and anti-His 1:200 (H-3, sc-8036) from Santa Cruz Biotechnology Inc (Dallas, TX, USA) was used. In the western blots anti-pp38 (D-8), anti-p38 (C-20), anti-ERK (C-9), anti-IκBα (H-4) from Santa Cruz Biotechnology Inc all used at 1:200 (Dallas, TX, USA), anti-pERK1/2 (T202), anti-pAkt (D9E), anti-Akt (40D4), from Cell Signaling Technology all used at 1:1000 (Leiden, Netherlands) were used. Full size western blot images are shown in Supplementary Figs. 6 and 7.

### Cell culture

Human monocyte THP1- Blue™ NFκB cells with a stably integrated NFκB-inducible SEAP reporter construct and THP1-Dual™ KO-MyD (Invivogen) for MyD88-independent IRF (luciferase) and NFκB (SEAP) pathways, normal THP1 cells (ATCC^®^ TIB-202^TM^, ATCC, LGC Standards, Middlesex, UK), and murine RAW264.7 macrophages (ATCC^®^- TIB-71^TM^) were cultured in RPMI-1640 and DMEM-high glucose medium (HyClone, GE Healthcare Life Science Laboratories), respectively, supplemented with 10% fetal bovine serum (Saveen Werner, Limhamn, Sweden). In experiments involving primary monocytes, the cells were cultured in OptiMEM (Gibco, Thermo Fischer) supplemented with 1% penicillin (100 U/ml) and streptomycin (100 μg/ml) (MP Biomedicals, Solon, OH, USA). Cells stimulated under serum-free conditions were washed and incubated for 2 h before treatment. For serum incubations, THP1-Blue™ NFκB and RAW264.7 cells were incubated in a medium containing 10% fetal bovine serum, while primary monocytes were incubated in a medium containing 10% human serum. Monocytes were differentiated to M1 macrophages using rhGM-CSF for 5 days, and subsequently treated with rhIFN-γ and LPS (*S*. Typhimurium), rhHMGB1, or rhS100A9 for additional 2 days. During the experiment, rWnt5a was added on days 1 and 3. Cells were collected on day 7 using non-enzymatic cell dissociation solution (Sigma–Aldrich).

### NFκB activity analysis

To determine NFκB activity, a colorimetric enzyme assay was performed. Sample absorbance was measured at OD_635_ nm in QUANTI-Blue medium (Invivogen) to detect and quantify secreted embryonic alkaline phosphatase (SEAP) secreted by the THP1-Blue™ NFκB cells, according to the manufacturer’s instructions.

### Quantitative real-time reverse-transcription PCR (qRT-PCR)

Total RNA was isolated using TRIzol reagent according to the manufacturer’s instructions (Invitrogen, Thermo Scientific). Then, cDNA was synthesized using random hexamer primers and MuLV reverse-transcriptase enzyme (Thermo Scientific). RT-qPCR was performed using Maxima SYBR Green/Rox reagents (Thermo Scientific) and the Mx3005P RT-qPCR system (Agilent Technologies, Santa Clara, CA, USA). The relative mRNA levels obtained using the comparative Ct method, and were normalized to *GAPDH*, *SDHA*, and *ACTB* expression^47^. Primer sequences are provided in Supplementary Table 1.

### Gene expression analysis

Gene expression of *Wnt5a* in primary macrophages upon endotoxin exposure in vivo was analysed using the publicly available microarray profile sets [GenBank: GPL570, 213425_at (ID_REF), GDS4419, 7474 (Gene ID), AI968085]^29^ from NCBI Gene Expression Omnibus profiles^30^.

### Flow cytometry

The cells were washed in flow cytometry buffer and stained with the antibodies indicated below for flow cytometry analysis. The cells were analysed using a lyse-wash protocol on FACS Verse (BD Biosciences, San Jose, CA, USA) with 7-aminoactinomycinD (7AAD) as a dead exclusion stain (BD Biosciences). The antibodies used were FITC-CD14 clone M5E2, PE-CD86 clone IT2.2, PeCy7-CD11c clone B-ly6, and APC-HLA-DR clone L243; all antibodies were from BD Biosciences.

### Surface plasmon resonance (SPR) analysis

For the experiments, 10 μg of rWnt5a or rWnt3a lyophilized from a solution in PBS, EDTA, and CHAPS, were reconstituted in 263 μl of HBS-P (10 mM HEPES, 0.15 M NaCl, and 0.005% surfactant P20, pH 7.4) to a final concentration of 1 μM (38 μg protein/ml). Then, 25 μl of each protein solution was diluted 10-fold by mixing with 175 μl of HBS-P buffer and 50 μl of 5x assay buffer (HBS-P with 100 μM ZnCl_2_ and 5 mM CaCl_2_). The samples were further diluted 1:1 by mixing 125 μl protein and 125 μl assay buffer in two steps, or for an analyte (protein) concentration range of 25–100 nM (~1–4 μg/ml). SPR analysis was performed using the Biacore 3000 system (GE Healthcare, Uppsala, Sweden). For the analysis, CM5 chip with an amine-coupled rhTLR2 in flow cell (Fc) 2 (immobilization level 4.0 kRU), rhTLR4/MD2 in Fc 3 (4.7 kRU), and rhTLR4 in Fc 4 (4.0 kRU), with Fc 1 as a reference surface was used. The samples were injected for 2 min at a rate of 30 μl/min in the assay buffer; the surface was regenerated by an injection of 15 μl of 3 mM EDTA in HBS-P followed by an injection of 15 μl of 10 mM glycine-HCl, pH 3.0. The resulting sensorgrams were fit to a 1:1 model using BIAevaluation 4.1 software (Biacore; GE Healthcare, Chicago, I, USA) to calculate the steady-state response (Req), which was then plotted against analyte concentration and fit to a one-site`hyperbola model to estimate the steady-state affinity (KD) and maximal response level (Bmax). All reagents were from R&D Systems (Minneapolis, MN, USA).

### In vitro protein binding assay

Proteins rhTLR4 and rWnt5a were mixed at a 1:2 molar ratio, and incubated in a binding buffer [10 mM HEPES (pH 7.9), 50 mM KCl, 0.2 mM EDTA, 1 mM DTT, 2.5 mM MgCl_2_, 10% glycerol, and 0.05% NP-40] for 30 min on ice. They were then separated by 7% native polyacrylamide gel electrophoresis. The proteins were either stained using Pierce silver stain kit (Thermo Fisher), or transferred onto a polyvinylidene fluoride membrane, and subsequently blotted using anti-Wnt5a (AF645; dilution 1:2000; R&D) and anti-His antibodies (Santa Cruz Biotechnology).

### Liquid chromatography-mass spectrometry (LC-MS/MS) analysis

In gel digestion was performed according to Shevchenko et al.^48^. The LC-MS/MS detection was performed using Tribrid mass spectrometer Fusion equipped with a Nanospray Flex ion source and coupled with an EASY-nLC 1000 pump (Thermo Fischer, Waltham, MA, USA). Peptides were concentrated on an Acclaim PepMap 100 C18 precolumn (75 μm x 2 cm, Thermo Fisher) and then separated on an Acclaim PepMap RSLC column (75 μm x 25 cm, nanoViper, C18, 2 μm, 100 Å), both heated at 40 °C. Solvent A (0.1% formic acid) and solvent B (0.1% formic acid in acetonitrile) were used to create a nonlinear gradient to elute the peptides. For the gradient, the percentage of solvent B increased from 5 to 22% in 20 min; then increased to 32% in 5 min; and finally increased to 90% in 2 min, which was maintained for a further 8 min to wash the columns.

Orbitrap Fusion mass spectrometer was operated in data-dependent acquisition mode. The peptides were introduced into the mass spectrometer via a stainless steel Nano-bore emitter (OD 150 μm, ID 30 μm) with the spray voltage of 2 kV and the capillary temperature set to 275 °C. Full MS survey scans from m/*z* 350–1350 with a resolution of 60,000 were performed in the Orbitrap detector. The automatic gain-control target was set to 4 x 10^5^ with an injecting time of 50 ms. The most intense ions (up to 20) with a charge state 2–5 after the full-scan MS were selected for fragmentation in Orbitrap. MS2 precursors were isolated using a quadrupole mass filter set to a width of 1.6 m/*z*. Precursors were fragmented by collision-induced dissociation and detected in an ion trap detector with a rapid scan rate. The collision energy for collision-induced dissociation was 27%. The resolution was set at 15,000, and the values for the automatic gain-control target and inject time were 2 x 10^3^ and 60 ms, respectively for MS/MS scans. The duration of dynamic exclusion was set to 30 s and the mass tolerance window to 10 ppm. MS/MS data were acquired in centroid mode. MS/MS spectra were searched with PEAKS (version 7.5) against UniProt Homo Sapiens (version 2016_05). A 5 ppm precursor tolerance and 0.02 Da fragment tolerance were used as the MS settings. Trypsin was selected as enzyme with two missed cleavage allowance, methionine oxidation and deamidation of aspargine and glutamine were treated as dynamic modification and carbamidomethylation of cysteine as a fixed modification. Maximum of post-translational modifications (PTM) per peptide was 2. Search results were filtered using 1% false discovery rate (FDR) and 2 unique peptides.

### Protein–protein docking

The crystal structure of the extracellular domain of the *Drosophila* Toll receptor corresponding to 201 N-terminal residues (28–228) fused to a fragment from the variable lymphocyte receptor (131–201) was used as a basis for the docking (pdb id 4arn). The Toll receptor was assumed to interact with WntD as a homodimer, and a homodimeric version of the Toll receptor was constructed from a dimer observed in the 3D crystal. Coordinates from WntD was taken from a crystal structure with pdb id 4krr that has 210 residues in the structure (residue 31–240). Before docking, the side-chains were repacked with the prepacking protocol in Rosetta macromolecular modelling package^49^. Global all-atom protein–protein docking generating 124,000 complex models was carried out with RosettaDock^49^, which simultaneously optimizes rigid body and side chain degrees of freedom during docking. The 200 lowest energy-docking models were analysed with structural clustering analysis to identify common docking poses and the lowest energy model was further studied by docking perturbation simulation that was started from the lowest energy-docking model rather than random rigid body orientation as in the global docking. Finally, local refinement docking, which starts from an existing docking model and skips the low-resolution search phase of RosettaDock was used to optimize the energy of the complex. Results were summarized in energy landscapes from the docking perturbation simulations starting from the lowest energy model identified in the energy refinement. In this plot the energy of models is plotted against root mean square deviation (rmsd) against the lowest energy model (interface rmsd).

### Statistics and reproducibility

Graph Pad Prism 7 software (La Jolla, CA, USA) was used for the statistical analyses. As indicated in figure legends, ordinary one-way analysis of variance (ANOVA) with multiple comparison tests, Dunnett’s or Dunn’s tests (solid lines in figures), Holm-Sidak´s test (grey annotations), or non-parametric Mann–Whitney U Wilcoxon test or student *t*-test (line with brackets in figures) were used. The significance threshold was set at *P* < 0.05. The experiments are presented as mean of at least three experiments and representative images are shown for blots and immunofluorescence.

### Reporting summary

Further information on research design is available in the Nature Research Reporting Summary linked to this article.

## Supplementary information

Supplementary Information

Description of Supplementary Data

Supplementary Data 1

Supplementary Data 2

Reporting Summary

## Data Availability

Gene expression data were analysed, using the publicly available microarray profile sets [GenBank: GPL570, 213425_at (ID_REF), GDS4419, 7474 (Gene ID), AI968085]^29^ from NCBI Gene Expression Omnibus profiles^30^. All datasets generated in the course of the current study are presented in the main text and the Supplementary Information. The source data underlying the Fig. 1 and Fig. 2 are presented in Supplementary Data 1 and 2, respectively.
