# Supplementary material for: Wnt5a is a TLR2/4-ligand that induces tolerance in human myeloid cells
Source: Commun Biol. 2019 May 9;2:176. doi: 10.1038/s42003-019-0432-4 (PMC6509336; doi:10.1038/s42003-019-0432-4)
Supplement: Supplementary file 1 — Supplementary Information [file 42003_2019_432_MOESM1_ESM.pdf]

Supplementary Figure 1. Wnt5a induces a Mo-MDSC–like CD14^{+}HLA-DR$^{low/-}$ CD86$^{low/-}$ cell population. **(a)** LPS induces *Wnt5a* expression, and Wnt5a induces *IL10* expression in primary human monocytes in non-endosomal MyD88-dependent pathways. (n=9) Dunnette's test was used for multiple comparisons. (**b**, **c**) Wnt5a induces Mo-MDSC–like cells together with PAMPs (LPS) (**a**) and DAMPs (HMGB1 or S100A9). (**c**) The CD14^{+}HLA-DR$^{low/-}$ cell populations in primary human monocyte cultures (determined by flow cytometry; red gates) are shown. PBMCs of healthy controls were differentiated into M1 macrophages using LPS, HMGB1, or S100A9 for 7 d in the presence of Wnt5a. On days 3 and 5, rWnt5a was added to induce Mo-MDSC–like cells [13], alone or in combination with the indicated molecules. (**d**) Wnt5a induces CD14^{+} HLA-DR$^{low/-}$ and CD14^{+} CD86$^{low/-}$ cells together with PAMPs and DAMPs. Percentage of CD14^{+} HLA-DR$^{low/-}$ (left) and CD14^{+} CD86$^{low/-}$ (right) cell populations in primary human monocytes cultures, as analysed by flow cytometry, is shown. The experiment was performed as in (**b, c**). Error bars indicate SEM (n=5); *$p<0.05$, **$p<0.01$, and ***$p<0.001$. Mann-Whitney U test was used for each pair.

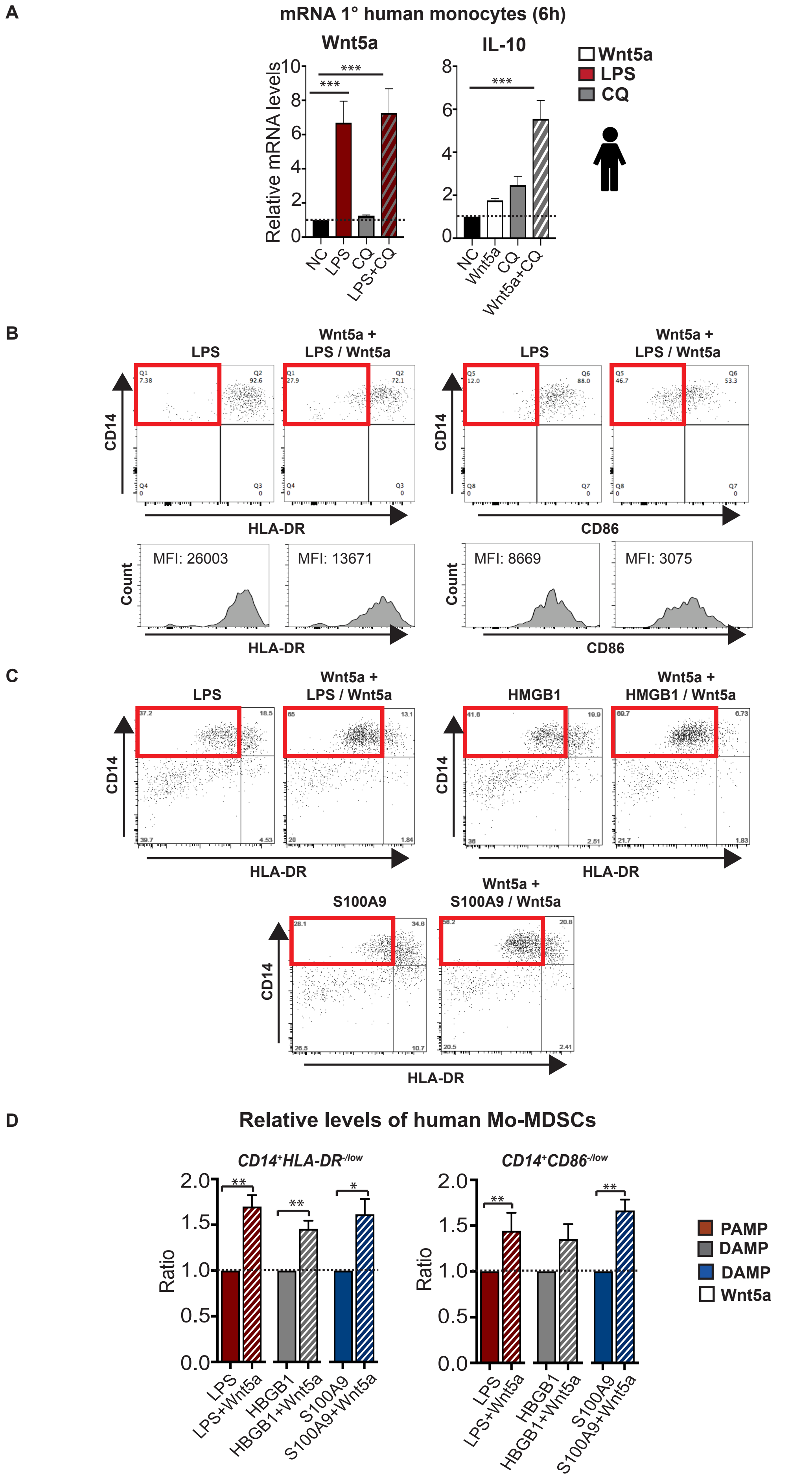
A
mRNA 1° human monocytes (6h)
Wnt5a
IL-10
Relative mRNA levels
NC LPS CQ LPS+CQ
NC Wnt5a CQ Wnt5a+CQ
Wnt5a
LPS
CQ
B
LPS
Wnt5a + LPS / Wnt5a
CD14
HLA-DR
CD86
MFI: 26003
MFI: 13671
MFI: 8669
MFI: 3075
Count
C
HMGB1
Wnt5a + HMGB1 / Wnt5a
S100A9
Wnt5a + S100A9 / Wnt5a
D
Relative levels of human Mo-MDSCs
CD14+HLA-DR-/low
CD14+CD86-/low
Ratio
LPS LPS+Wnt5a HBGB1 HBGB1+Wnt5a S100A9 S100A9+Wnt5a
PAMP
DAMP
DAMP
Wnt5a

Supplementary Figure 2. Wnt5a does not induce pro-inflammatory cytokines in primary human monocytes. (**a**) Wnt5a does not induce pro-inflammatory cytokines in primary human monocytes after short-term incubation. Primary human monocytes from healthy controls were treated with rWnt5a and LPS for 6 h in the absence of serum. Levels of secreted IL-1β and IL-12 were analysed by CBA (left) and PGE2 by ELISA (right). (n=5 and n=3). Dunn's test was used for multiple comparisons. (**b**) CBA analysis of Wnt5a induced cytokines after long-term incubation. The experiment was performed as in (**a**), except that cytokine levels were analysed after 24 h. Levels of secreted cytokines were analysed by CBA. (n=3). Dunn's test was used for multiple comparisons. (**c**) Monocytes from healthy controls were treated with rWnt5a and LPS for 24 h in the absence of serum, and *IL10* levels were analysed by RT-qPCR. (n=3) Dunn's test was used for multiple comparisons. (**d**) Wnt5a induces cytokine production in primary human monocytes in the presence of serum. The experiment was performed as in (**a**) but in the presence of human serum. (n=3) Dunn's test was used for multiple comparisons. (**e**) The rWnt5a and rWnt3a preparations used in the current study are endotoxin-free. Endotoxin levels in the protein preparations were determined using the *Limulus* endotoxin assay. Black bars, standard titration of endotoxin solutions provided by the manufacturers; white bars, endotoxin levels in two separate batches of rWnt5a and rWnt3a preparation compared to the manufacturers´ standard solutions (negative, <0.005 EU/ml). (**f**) Wnt5a does not induce NFκB activity. THP-1- Blue™ NFκB cells with a stably integrated NFκB-inducible SEAP reporter construct were treated with rWnt5a and/or LPS (12 and 24 h). (n=4). Dunn's test was used for multiple comparisons. (**g**) A DAMP, S100A9, induces NFκB activity. THP-1- Blue™ NFκB cells with a stably integrated NFκB-inducible SEAP reporter construct were treated with rWnt5a, LPS or S100A9 (6 h). (n=3) Dunnett's test was used for multiple comparisons. Error bars indicate SEM; *p<0.05, **p<0.01, and ***p<0.001. Data were analysed by ANOVA or t-test as indicated.

S Fig. 2

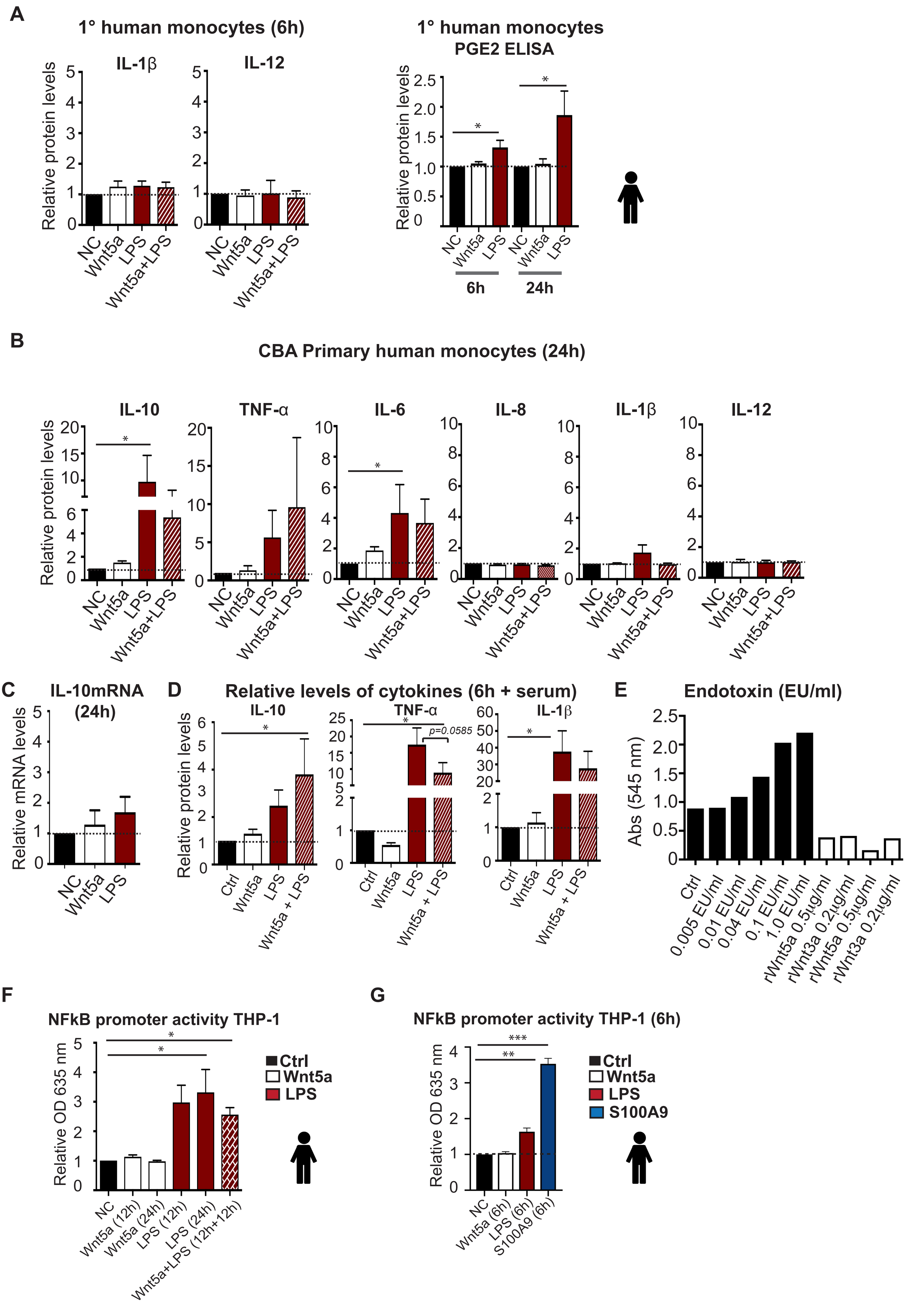
A
1° human monocytes (6h)
IL-1β
IL-12
Relative protein levels
NC
Wnt5a
LPS
Wnt5a+LPS
1° human monocytes
PGE2 ELISA
6h
24h
B
CBA Primary human monocytes (24h)
IL-10
TNF-α
IL-6
IL-8
IL-1β
IL-12
C
IL-10mRNA (24h)
Relative mRNA levels
D
Relative levels of cytokines (6h + serum)
Ctrl
Wnt5a + LPS
p=0.0585
E
Endotoxin (EU/ml)
Abs (545 nm)
0.005 EU/ml
0.01 EU/ml
0.04 EU/ml
0.1 EU/ml
1.0 EU/ml
rWnt5a 0.5μg/ml
rWnt3a 0.2μg/ml
rWnt5a 0.5μg/ml
rWnt3a 0.2μg/ml
F
NFkB promoter activity THP-1
Relative OD 635 nm
Wnt5a (12h)
Wnt5a (24h)
LPS (12h)
LPS (24h)
Wnt5a+LPS (12h+12h)
G
NFkB promoter activity THP-1 (6h)
Wnt5a (6h)
LPS (6h)
S100A9 (6h)
S100A9

Supplementary Figure 3.

(a) Western blots of THP-1 cells transfected with HA-Wnt5a or Ctrl-anti-sense-Wnt5a were stimulated with LPS during a time-course and subjected to western blot to investigate the phosphorylation status of p38, ERK1/2, Akt and presence of IκBα. One representative experiment is shown. **(b)** Western blots of primary human monocytes stimulated with rWnt5a during a time-course and subjected to western blot to investigate the phosphorylation status of p38, ERK1/2, Akt. One representative experiment is shown.

S Figure 3

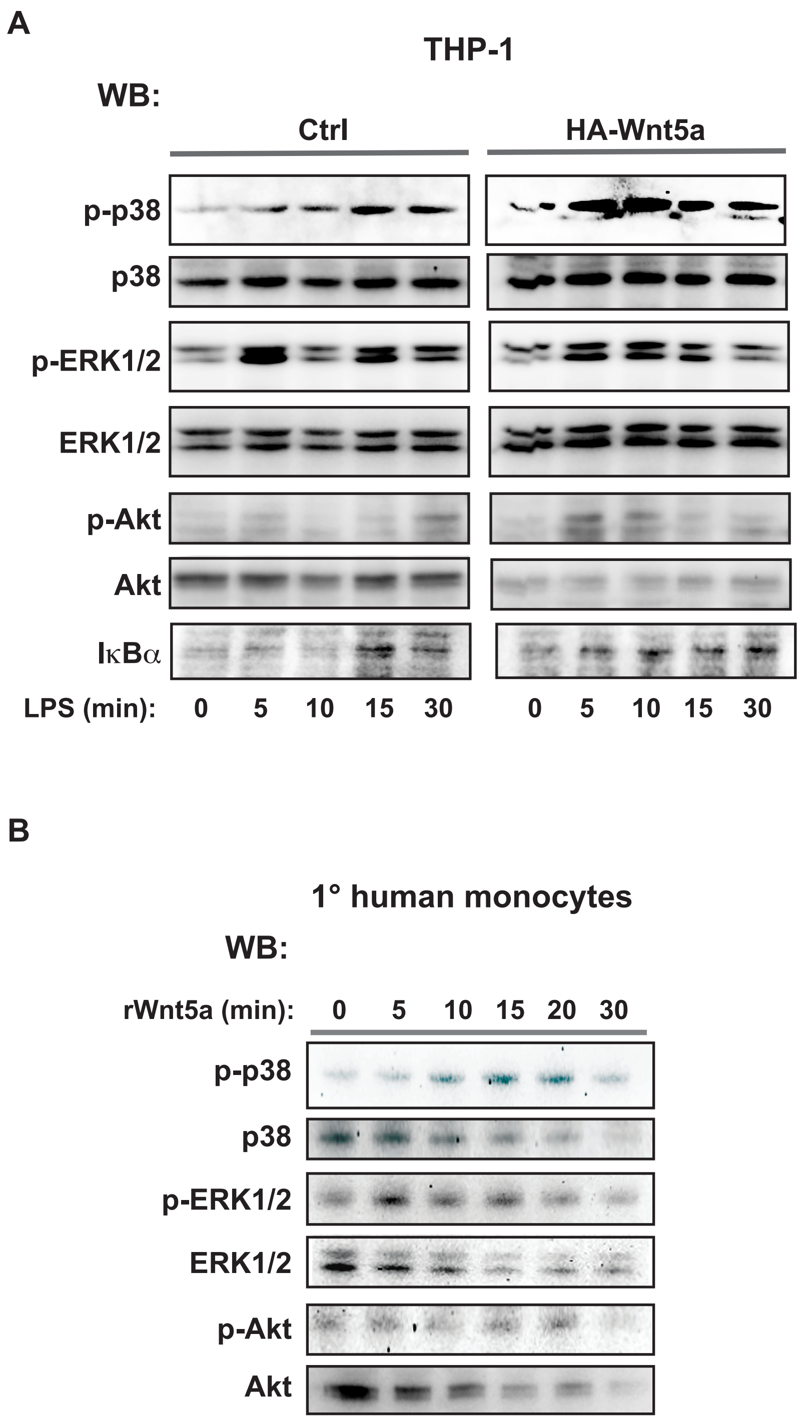
A
THP-1
WB:
Ctrl
HA-Wnt5a
p-p38
p38
p-ERK1/2
ERK1/2
p-Akt
Akt
IκBα
LPS (min): 0 5 10 15 30 0 5 10 15 30
B
1° human monocytes
WB:
rWnt5a (min): 0 5 10 15 20 30
p-p38
p38
p-ERK1/2
ERK1/2
p-Akt
Akt

Supplementary Figure 4. (**a**) LPS induced cytokine production in mouse cells. Murine RAW264.7 macrophages (left), or primary WT C57Bl/6 BMM, *MyD88*$^{-/-}$ BMM, or *Tlr4*$^{-/-}$ BMM (right), were treated with LPS for 6 h. IL-6, IL-10, TNFα, MCP1, IFN-γ and IL-12 cytokine levels were measured by cytokine bead array (CBA) analysis. (n=3 and n=4). Dunn's test was used for multiple comparisons. The Wnt5a stimulation data are shown in Figure 2. (**b**) Wnt5a induces MCP-1(CCL2) through Fz/Dvl in mouse RAW264.7 macrophages. Addition of two Dishevelled (Dvl) inhibitors (NCS and Dvl) significantly inhibited the Wnt5a-induced production of MCP-1 (Dvl), but not that of TNFα in mouse RAW264.7 macrophages. (n=3) Dunn's test was used for multiple comparisons. Error bars indicate SEM; *p<0.05, **p<0.01, and ***p<0.001. Data were analysed by ANOVA or t-test as indicated.

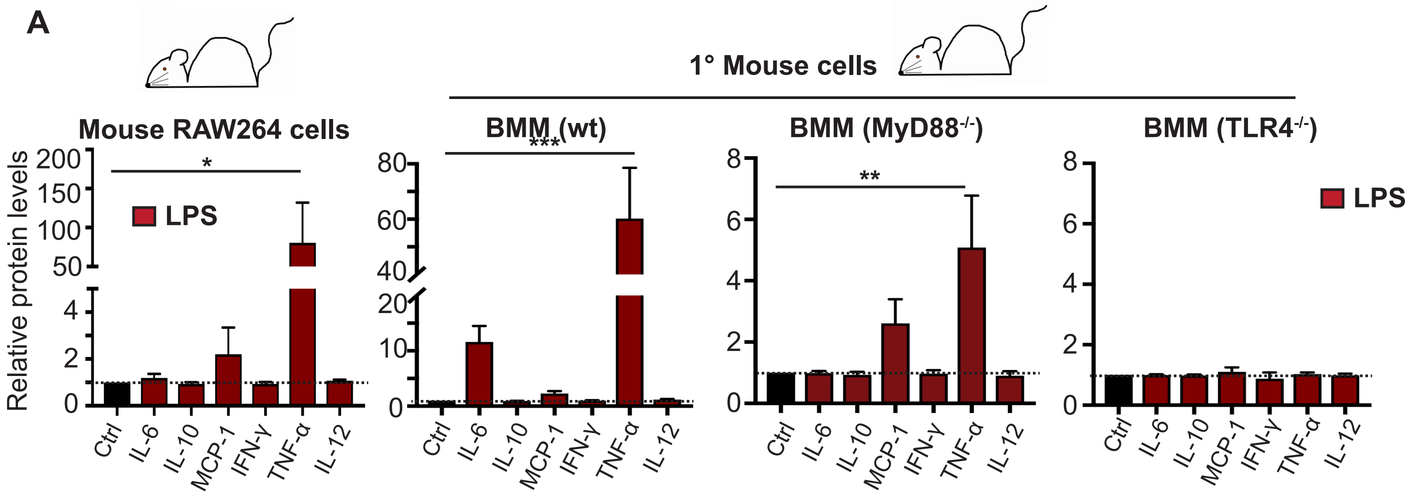
A
1° Mouse cells
Mouse RAW264 cells
BMM (wt)
BMM (MyD88-/-)
BMM (TLR4-/-)
Relative protein levels
LPS
LPS
*
***
**
Ctrl
IL-6
IL-10
MCP-1
IFN-γ
TNF-α
IL-12

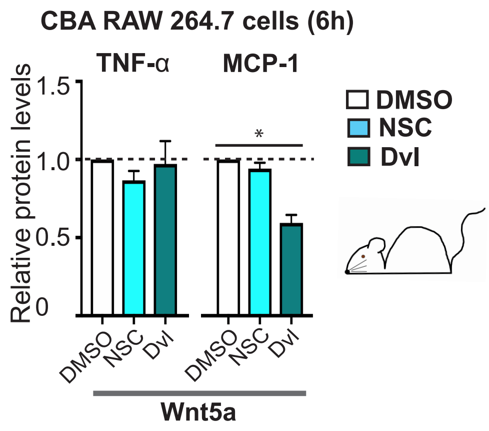
B
CBA RAW 264.7 cells (6h)
TNF-α
MCP-1
Relative protein levels
DMSO
NSC
Dvl
*
Wnt5a

Supplementary Figure 5. Wnt5a can signal through TLR4/MyD88. (**a**) THP-1 cells after MyD88 inhibition. THP-1 cells overexpressing HA-Wnt5a were treated with a MyD88 inhibitor (MyD88 inh) or control inhibitor peptide (Ctrl inh). Relative protein levels of secreted cytokines are shown. (n=5) Dunn's test was used for multiple comparisons. (**b**) The effect of rWnt5a on IL-10 induction is not inhibited by TLR2 and TLR4-blocking antibodies. Primary human monocytes were pre-treated for 1 h with Ctrl-pAb, TLR2-pAb or TLR4-pAb (n=4), and subsequently stimulated with either rWnt5a or LPS for 6 h. IL-10 levels were determined using ELISA. Holm-Sidak's test was used for multiple comparisons. (**c**) Wnt5a signalling in human primary monocytes is affected by PMB. The relative protein levels of IL-8 (determined by ELISA), and IL-1β, IL-6, IL-10, TNFα, and IL-12 levels (determined by CBA) secreted by primary human monocytes stimulated with rWnt5a (6 h), and treated or not with PMB are shown. The ratio compared to Ctrl (black bars). (n=4) Dunn's test was used for multiple comparisons. (n=4) Mann-Whitney U test was used for each pair comparison. (**d**) A dose range experiment investigating Wnt5a induced expression of *IL10* mRNA in primary human monocytes was performed to show that rWnt5a was used at the proper concentration (0.5µg/ml). A boiled rWnt5a sample was added at the same concentration to show lack of biological effects. (n=6) Dunnett's test was used for multiple comparisons. Error bars indicate SEM; *$p<0.05$, **$p<0.01$, and ***$p<0.001$. Data were analysed by ANOVA or t-test as indicated.

S Figure 5

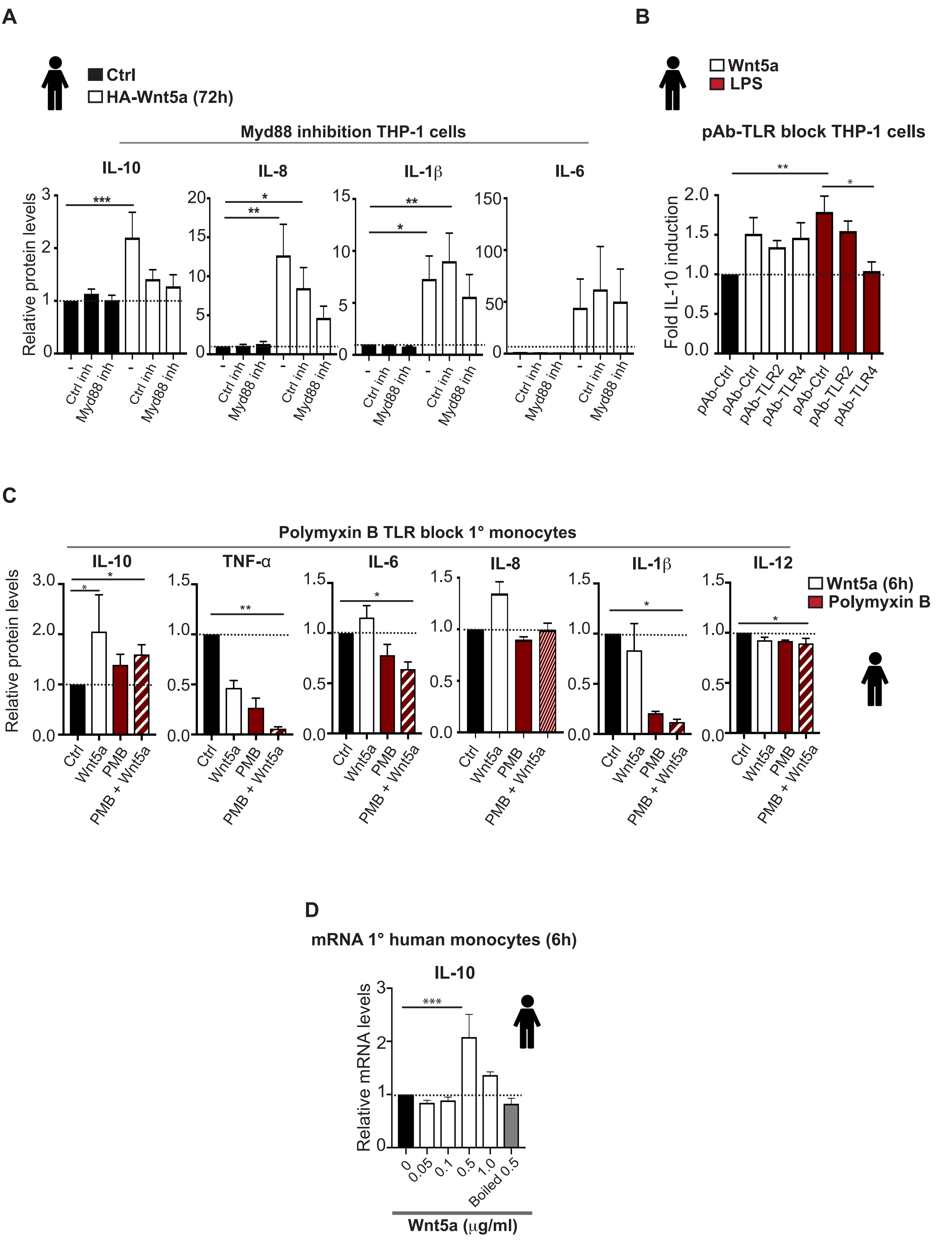
A
Ctrl
HA-Wnt5a (72h)
Myd88 inhibition THP-1 cells
IL-10
IL-8
IL-1β
IL-6
Relative protein levels
Ctrl inh
Myd88 inh
B
Wnt5a
LPS
pAb-TLR block THP-1 cells
Fold IL-10 induction
pAb-Ctrl
pAb-TLR2
pAb-TLR4
C
Polymyxin B TLR block 1° monocytes
IL-10
TNF-α
IL-6
IL-8
IL-1β
IL-12
Wnt5a (6h)
Polymyxin B
Relative protein levels
Ctrl
Wnt5a
PMB
PMB + Wnt5a
D
mRNA 1° human monocytes (6h)
IL-10
Relative mRNA levels
Boiled 0.5
Wnt5a (μg/ml)

Supplementary Figure 6. Full western blot images of Figure 1A and Supplementary Figure 3A.

S Fig 6

Raw data Figure 1A

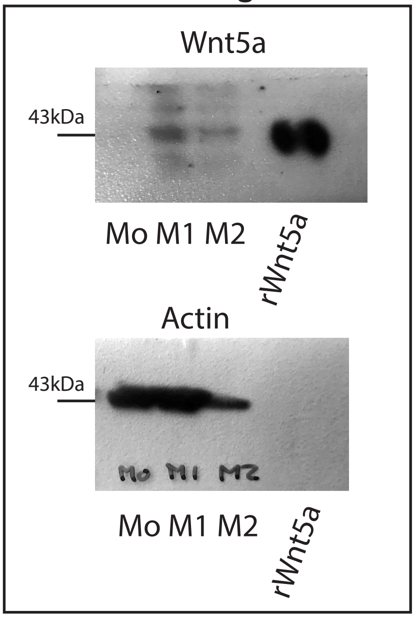

Raw data Supplementary Figure 3A

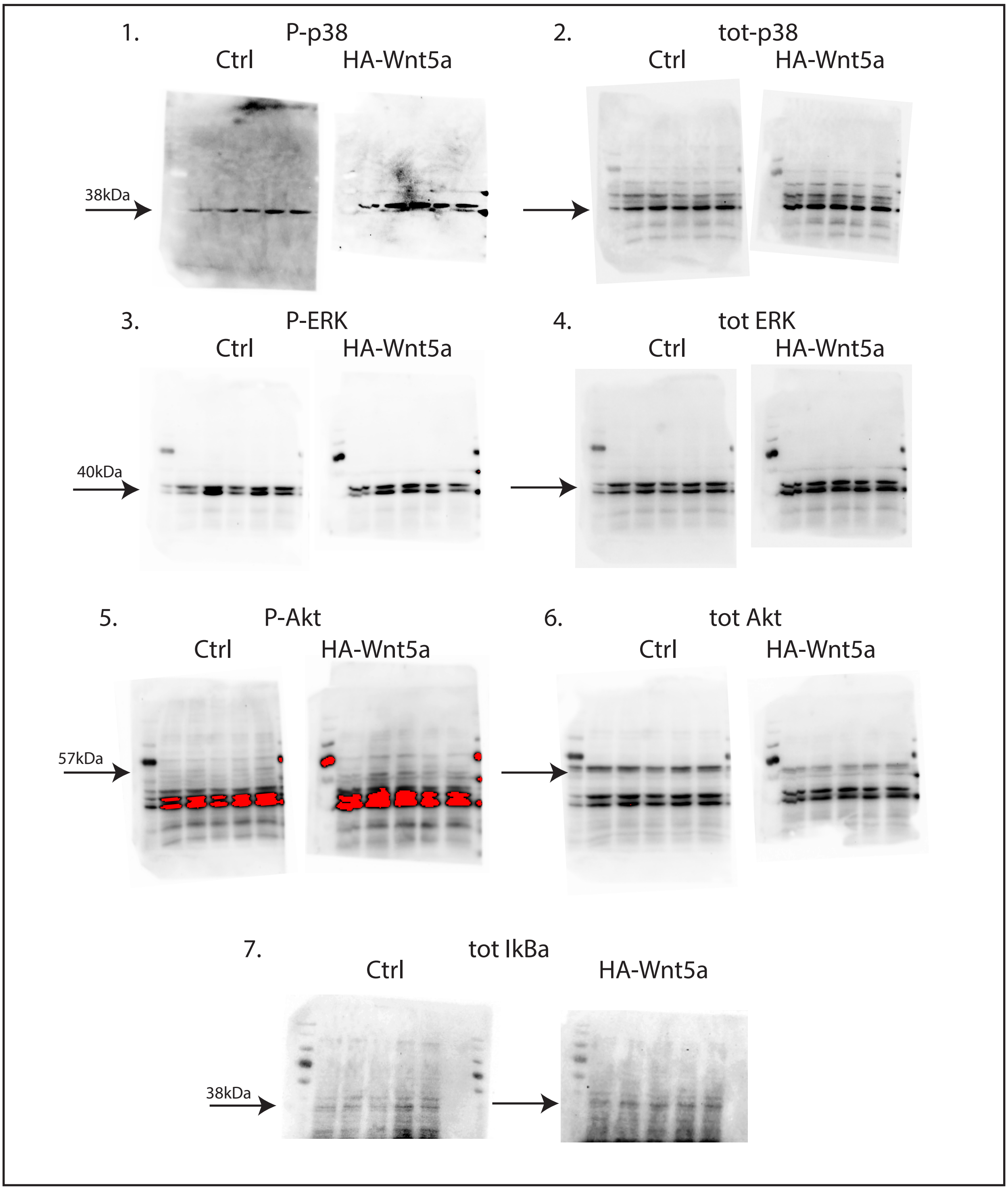

Supplementary Figure 7. Full western blot images of Supplementary Figure 3B.

Raw data Supplementary Figure 3B

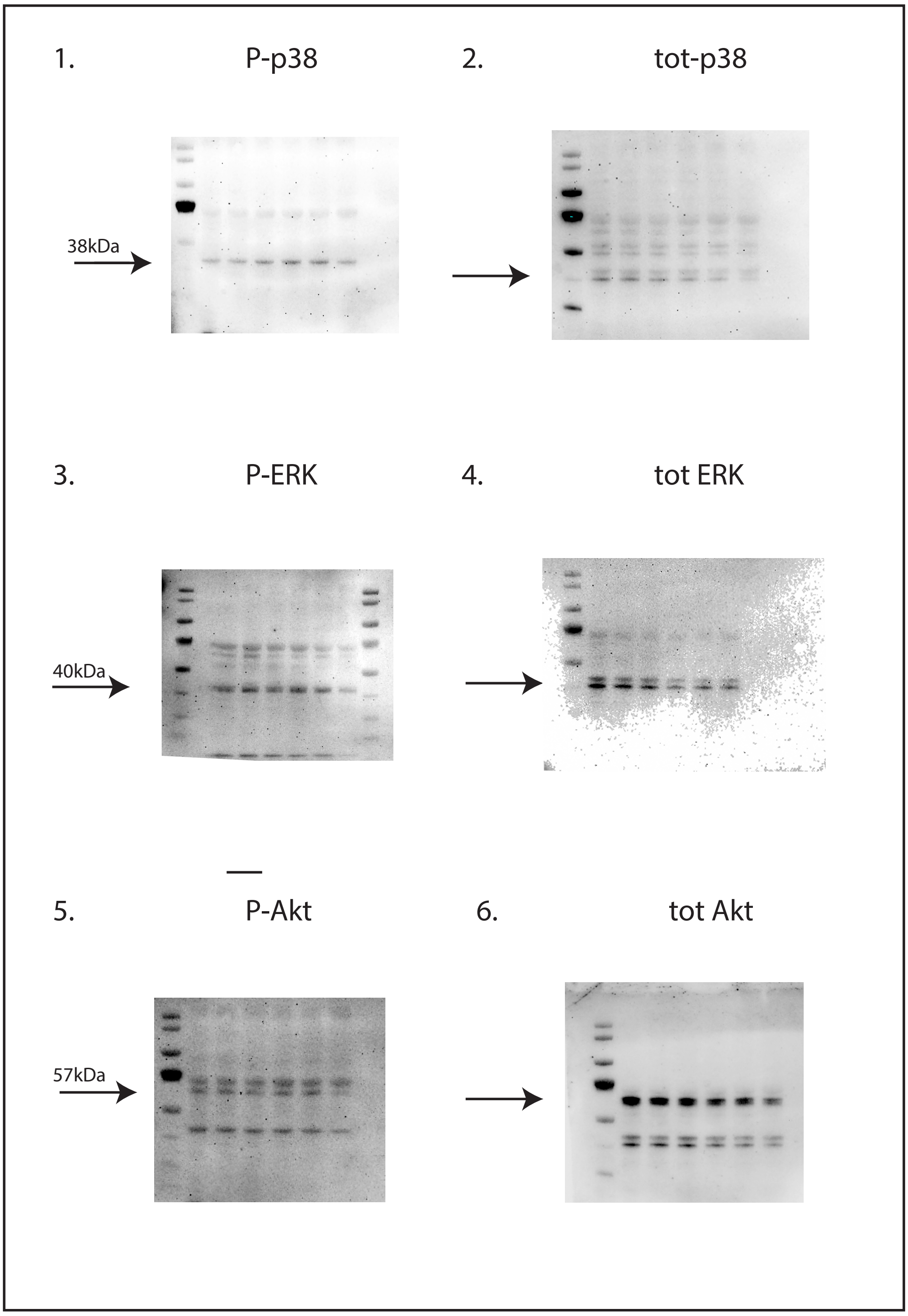

Supplementary Table 1

Primers used in the current study.

Primer	Sequence (5′–3′)[a]
SDHA	**F:** TGGGAACAAGAGGGCATCTG, **R:** CCACCACTGCATCAAATTCATG
GAPDH	**F:** TGCACCACCAACTGCTTAGC, **R:** TGGCATGGACTGTGGTCATGAG
ACTB	**F:** CTGGAACGGTGAAGGTGACA, **R**: AAGGGACTTCCTGTAACAATGCA
IL-6	**F:** GGCACTGGCAGAAAACAACC, **R:** GCAAGTCTCCTCATTGAATCC
IL-8	**F:** ACTGAGAGTGATTGAGAGTGGAC, **R:** AACCCTCTGCACCCAGTTTTC
IL-10	**F:** CCCTGGGTGAGAAGCTGAAG, **R:** CACTGCCTTGCTCTTATTTTCACA
TNF	**F:** GCAGGTCTACTTTGGGATCATTG, **R:** GCGTTTGGGAAGGTTGGA
WNT5A	**F**: AGGGCTCCTACGAGAGTGCT, **R**: GACACCCCATGGCACTTG

[a]F, forward; R, reverse

Supplementary Table 2

LC-MS/MS analysis of rWnt5a confirming the purity of the protein preparation.

Accession	-10lgP	Coverage (%	Area Sampl	#Unique pe	Avg. Mass	Description
P02768|ALBU_HUMAN	137,04	7	2,46E+09	7	69367	Serum albumin OS=Homo sapiens OX=9606 GN=ALB PE=1 SV=2
P41221|WNT5A_HUMAN	186,64	27	6,73E+08	15	42340	Protein Wnt-5a OS=Homo sapiens OX=9606 GN=WNT5A PE=1 SV=2
Q92626|PXDN_HUMAN	198,69	12	5,40E+07	15	165274	Peroxidasin homolog OS=Homo sapiens OX=9606 GN=PXDN PE=1 SV=2
Q92743|HTRA1_HUMAN	137,25	28	3,73E+07	8	51287	Serine protease HTRA1 OS=Homo sapiens OX=9606 GN=HTRA1 PE=1 SV=1
P04406|G3P_HUMAN	128,57	18	3,50E+07	4	36053	Glyceraldehyde-3-phosphate dehydrogenase OS=Homo sapiens OX=9606 GN=GAPDH PE=1 SV=3
Q9BXJ3|C1QT4_HUMAN	148,88	34	3,39E+07	6	35256	Complement C1q tumor necrosis factor-related protein 4 OS=Homo sapiens OX=9606 GN=C1QTNF4 PE=1 SV=2
Q14103|HNRPD_HUMAN	141	16	3,12E+07	6	38434	Heterogeneous nuclear ribonucleoprotein D0 OS=Homo sapiens OX=9606 GN=HNRNPD PE=1 SV=1
Q99542|MMP19_HUMAN	113,92	15	2,32E+07	4	57357	Matrix metalloproteinase-19 OS=Homo sapiens OX=9606 GN=MMP19 PE=1 SV=1
P20290|BTF3_HUMAN	112,61	33	1,92E+07	3	22168	Transcription factor BTF3 OS=Homo sapiens OX=9606 GN=BTF3 PE=1 SV=1
E9PAV3|NACAM_HUMAN	103,81	4	1,53E+07	5	205419	Nascent polypeptide-associated complex subunit alpha muscle-specific form OS=Homo sapiens OX=9606 GN=NACA PE=1 SV=1
Q13765|NACA_HUMAN	103,81	42	1,53E+07	5	23384	Nascent polypeptide-associated complex subunit alpha OS=Homo sapiens OX=9606 GN=NACA PE=1 SV=1
P14618|KPYM_HUMAN	158,51	19	1,31E+07	8	57937	Pyruvate kinase PKM OS=Homo sapiens OX=9606 GN=PKM PE=1 SV=4
P09651|ROA1_HUMAN	150,43	29	1,08E+07	9	38747	Heterogeneous nuclear ribonucleoprotein A1 OS=Homo sapiens OX=9606 GN=HNRNPA1 PE=1 SV=5
Q13303|KCAB2_HUMAN	120,43	18	8,55E+06	5	41000	Voltage-gated potassium channel subunit beta-2 OS=Homo sapiens OX=9606 GN=KCNAB2 PE=1 SV=2
Q86V81|THOC4_HUMAN	124,36	23	8,48E+06	6	26888	THO complex subunit 4 OS=Homo sapiens OX=9606 GN=ALYREF PE=1 SV=3
P23246|SFPQ_HUMAN	103,57	11	5,39E+06	6	76150	Splicing factor proline- and glutamine-rich OS=Homo sapiens OX=9606 GN=SFPQ PE=1 SV=2
P46783|RS10_HUMAN	113,31	30	4,67E+06	4	18898	40S ribosomal protein S10 OS=Homo sapiens OX=9606 GN=RPS10 PE=1 SV=1
P30419|NMT1_HUMAN	119,76	10	3,76E+06	4	56806	Glycylpeptide N-tetradecanoyltransferase 1 OS=Homo sapiens OX=9606 GN=NMT1 PE=1 SV=2
P46940|IQGA1_HUMAN	104,41	2	7,53E+05	3	189251	Ras GTPase-activating-like protein IQGAP1 OS=Homo sapiens OX=9606 GN=IQGAP1 PE=1 SV=1

Supplementary Table 3

LC-MS/MS analysis of a native gel band from the *in vitro* binding assay shown in Figure 4a. (**a**) The digested band contains TLR4 protein. Protein coverage, with the supporting peptides, is shown. (**b**) The digested band contains Wnt5a protein. Protein coverage, with the supporting peptides, is shown.

a. TL4R peptides identified by mass spectrometry highest PEAKS scores are given.

**Amino acid**	**Sequence**	**Score**	**Charge**	**Experimental weight**	**Theoretical weight**
58-67	NLDLSFNPLR	36	2+	1187.629	1187.630
58-67	**N**LDLSFNPLR	21	2+	1188.614	1188.614
131-150	LVAVETNLASLENFPIGHLK	23	2+	2164.186	2164.189
154-166	ELNVAHNLIQSFK	22	3+	1511.810	1511.810
187-196	IQSIYCTDLR	21	2+	1267.622	1267.623
235-244	NNFDSLNVMK	19	2+	1180.554	1180.555
235-244	**N**NFDSLNV**M**K	16	2+	1197.531	1197.534
245-257	TCIQGLAGLEVHR	24	2+	1452.749	1452.751
258-271	LVLGEFRNEGNLEK	25	3+	1616.854	1616.852
258-264	LVLGEFR	22	2+	832.480	832.481
342-349	FGQFPTLK	38	2+	936.506	936.507
356-382	LTFTSNKGGNAFSEVDLPSLEFL DLSR	26	3+	2956.482	2956.482
363-382	GGNAFSEVDLPSLEFLDLSR	23	3+	2165.070	2165.064
383-402	**N**GLSFKGCCSQSDFGTTSLK	37	2+	2193.963	2193.967
389-402	GCCSQSDFGTTSLK	19	3+	1546.638	1546.639
448-460	NLIYLDISHTHTR	15	3+	1581.826	1581.826
461-477	VAFNGIFNGLSSLEVLK	42	2+	1806.985	1806.988
461-477	VAF**N**GIF**N**GLSSLEVLK	17	2+	1808.962	1808.956
599-606	QLLVEVER	28	2+	984.560	984.560

Oxidized methionine and deamidated aspargine are denoted as bold letter. The highest PEAKS scores are given.

Identified peptides denoted in red

MMSASRLAGTLIPAMAFLSCVRPESWEPCVEVVPNITYQCMELNFYKIPDNLPFSTKNLD
LSFNPLRHLGSYSFFSFPELQVLDLSRCEIQTIEDGAYQSLSHLSTLILTGNPIQSLALG
AFSGLSSLQKLVAVETNLASLENFPIGHLKTLKELNVAHNLIQSFKLPEYFSNLTNLEHL
DLSSNKIQSIYCTDLRVLHQMPLLNLSLDLSLNPMNFIQPGAFKEIRLHKLTLRNNFDSL
NVMKTCIQGLAGLEVHRLVLGEFRNEGNLEKFDKSALEGLCNLTIEEFRLAYLDYYLDDI
IDLFNCLTNVSSFSLVSVTIERVKDFSYNFGWQHLELVNCKFGQFPTLKLKSLKRLTFTS
NKGGNAFSEVDLPSLEFLDLSRNGLSFKGCCSQSDFGTTSLKYLDLSFNGVITMSSNFLG
LEQLEHLDFQHSNLKQMSEFSVFLSLRNLIYLDISHTHTRVAFNGIFNGLSSLEVLKMAG
NSFQENFLPDIFTELRNLTFLDLSQCQLEQLSPTAFNSLSSLQVLNMSHNNFFSLDTFPY
KCLNSLQVLDYSLNHIMTSKKQELQHFPSSLAFLNLTQNDFACTCEHQSFLQWIKDQRQL
LVEVERMECATPSDKQGMPVLSLNITCQMNKTIIGVSVLSVLVVSVVAVLVYKFYFHLML
LAGCIKYGRGENIYDAFVIYSSQDEDWVRNELVKNLEEGVPPFQLCLHYRDFIPGVAIAA
NIIHEGFHKSRKVIVVVSQHFIQSRWCIFEYEIAQTWQFLSSRAGIIFIVLQKVEKTLLR
QQVELYRLLSRNTYLEWEDSVLGRHIFWRRLRKALLDGKSWNPEGTVGTGCNWQEATSI

b. Wnt5a peptides identified by mass spectrometry highest PEAKS scores are given.

Amino acid	Sequence	Score	Charge	Experimental weight	Theoretical weight
99-109	TGIKECQYQFR	25	3+	1428.677	1428.682
103-109	ECQYQFR	27	2+	1029.433	1029.434
156-166	EGELSTCGCSR	20	2+	1254.496	1254.497
195-200	EFVDAR	28	2+	735.354	735.355
229-239	TVYNLADVACK	22	2+	1252.612	1252.612
284-290	LVQV**N**SR	18	2+	815.448	815.450
343-348	GYDQFK	26	2+	756.343	756.344
343-354	GYDQFKTVQTER	18	3+	1470.710	1470.710
370-380	CTEIVDQFVCK	29	2+	1397.630	1397.632

Oxidized methionine is denoted as bold letter. The highest PEAKS scores are given.

Identified peptides denoted in red

MKKPIGILSPGVALGTAGGAMSSKFFLMALATFFSFAQVVIEANSWWSLGMNNPVQMSEVYIIGAQPLCS
QLAGLSQGQKKLCHLYQDHMQYIGEGAKTGIKECQYQFRHRRWNCSTVDNTSVFGRVMQIGSRETAFTYA
VSAAGVVNAMSRACREGELSTCGCSRAARPKDLPRDWLWGGCGDNIDYGYRFAKEFVDARERERIHAKGS
YESARILMNLHNNEAGRRTVYNLADVACKCHGVSGSCSLKTCWLQLADFRKVGDALKEKYDSAAAMRLNS
RGKLVQVNSRFNSPTTQDLVYIDPSPDYCVRNESTGSLGTQGRLCNKTSEGMDGCELMCCGRGYDQFKTV
QTERCHCKFHWCCYVKCKKCTEIVDQFVCK
