# Supplementary material for: Wnt5a is a TLR2/4-ligand that induces tolerance in human myeloid cells
Source: Commun Biol. 2019 May 9;2:176. doi: 10.1038/s42003-019-0432-4 (PMC6509336; doi:10.1038/s42003-019-0432-4)
Supplement: Supplementary file 2 — Description of Supplementary Data [file 42003_2019_432_MOESM2_ESM.pdf]

Description of Additional Supplementary Files

File Name: Supplementary Data 1
Description: Raw data Figure 1

File Name: Supplementary Data 2
Description: Raw data Figure 2
