# Supplementary material for: Wnt5a is a TLR2/4-ligand that induces tolerance in human myeloid cells
Source: Commun Biol. 2019 May 9;2:176. doi: 10.1038/s42003-019-0432-4 (PMC6509336; doi:10.1038/s42003-019-0432-4)
Supplement: Supplementary file 5 — Reporting Summary [file 42003_2019_432_MOESM5_ESM.pdf]

nature research

Corresponding author(s): Karin Leandersson
April 18, 2019

Reporting Summary

Nature Research wishes to improve the reproducibility of the work that we publish. This form provides structure for consistency and transparency in reporting. For further information on Nature Research policies, see Authors & Referees and the Editorial Policy Checklist.

Statistical parameters

When statistical analyses are reported, confirm that the following items are present in the relevant location (e.g. figure legend, table legend, main text, or Methods section).

n/a	Confirmed	
☐	☒	The exact sample size (*n*) for each experimental group/condition, given as a discrete number and unit of measurement
☐	☒	An indication of whether measurements were taken from distinct samples or whether the same sample was measured repeatedly
☐	☒	The statistical test(s) used AND whether they are one- or two-sided<br>*Only common tests should be described solely by name; describe more complex techniques in the Methods section.*
☒	☐	A description of all covariates tested
☐	☒	A description of any assumptions or corrections, such as tests of normality and adjustment for multiple comparisons
☐	☒	A full description of the statistics including central tendency (e.g. means) or other basic estimates (e.g. regression coefficient) AND variation (e.g. standard deviation) or associated estimates of uncertainty (e.g. confidence intervals)
☒	☐	For null hypothesis testing, the test statistic (e.g. *F*, *t*, *r*) with confidence intervals, effect sizes, degrees of freedom and *P* value noted<br>*Give P values as exact values whenever suitable.*
☒	☐	For Bayesian analysis, information on the choice of priors and Markov chain Monte Carlo settings
☒	☐	For hierarchical and complex designs, identification of the appropriate level for tests and full reporting of outcomes
☒	☐	Estimates of effect sizes (e.g. Cohen's *d*, Pearson's *r*), indicating how they were calculated
☐	☒	Clearly defined error bars<br>*State explicitly what error bars represent (e.g. SD, SE, CI)*

Our web collection on statistics for biologists may be useful.

Software and code

Policy information about availability of computer code

Data collection	All datasets generated in the course of the current study are presented in the main text and the Supplementary Information available online.<br>Gene expression data was analysed using the publicly available microarray profile sets (and therefore not ours and not presented online as commented on) GenBank: GPL570, 213425_at (ID_REF), GDS4419, 7474 (Gene ID), AI968085 28 from NCBI Gene Expression Omnibus profiles 29.
Data analysis	Graph Pad Prism 7 software (La Jolla, CA, USA)<br>www.rosettacommons.org

For manuscripts utilizing custom algorithms or software that are central to the research but not yet described in published literature, software must be made available to editors/reviewers upon request. We strongly encourage code deposition in a community repository (e.g. GitHub). See the Nature Research guidelines for submitting code & software for further information.

Data

Policy information about availability of data

All manuscripts must include a data availability statement. This statement should provide the following information, where applicable:

- Accession codes, unique identifiers, or web links for publicly available datasets
- A list of figures that have associated raw data
- A description of any restrictions on data availability

Data availability
All datasets generated in the course of the current study are presented in the main text and the Supplementary Information available online.
Gene expression data was analysed using the publicly available microarray profile sets (and therefore not ours and not presented online as commented on) GenBank: GPL570, 213425_at (ID_REF), GDS4419, 7474 (Gene ID), AI968085 28 from NCBI Gene Expression Omnibus profiles 29.
The protein structure data are from programming models and not novel experimentally solved structures but only modelling and Rosetta.org is referred to.

Field-specific reporting

Please select the best fit for your research. If you are not sure, read the appropriate sections before making your selection.

☒ Life sciences ☐ Behavioural & social sciences ☐ Ecological, evolutionary & environmental sciences

For a reference copy of the document with all sections, see nature.com/authors/policies/ReportingSummary-flat.pdf

Life sciences study design

All studies must disclose on these points even when the disclosure is negative.

Sample size	We aim at using N=5-10 as routine depending on if its primary cells or cell line experiments but N=3 is the least. Samples were collected from individual experiments from individual donors (mouse and human). For cell lines samples were collected from individual experiments. Two exceptions where two individual experiments were collected as controls due to endotoxin contamination checks on separate protein batches which is important to check for this study (endotoxin test of recombinant wnt used (Suppl Fig 2e) and Biacore analysis of recombinant wnt data (Table 1) now shown as separate bars in (Suppl Fig 2e).
Data exclusions	Data was only excluded if positive control did not work = experiment did not work = was never used in calculations ie. not excluded afterwards.
Replication	Three replicates at least unless otherwise stated.
Randomization	n/a no such experiment
Blinding	n/a no such experiment

Reporting for specific materials, systems and methods

Materials & experimental systems

n/a	Involved in the study	
☐	☒	Unique biological materials
☐	☒	Antibodies
☐	☒	Eukaryotic cell lines
☒	☐	Palaeontology
☐	☒	Animals and other organisms
☐	☒	Human research participants

Methods

n/a	Involved in the study	
☒	☐	ChIP-seq
☐	☒	Flow cytometry
☒	☐	MRI-based neuroimaging

Unique biological materials

Policy information about availability of materials

Obtaining unique materials	Recombinant human S100A9 (rhS100A9) was a gift from Active Biotech AB (Lund, Sweden). All other recombinant human compounds were purchased from R&D Systems (Minneapolis, MN, USA). recombinant human/

mouse (rh/m)Wnt5a, rmWnt3a (rWnt3a;), rhGM-CSF, rhIFN-γ, HMGB1, rhTLR4. LPS from Salmonella Typhimurium, polymyxin B (PMB), and actinomycin D (10 ?g/ml) were purchased from Sigma Aldrich. MyD88 inhibitor and control peptide set NBP2-29328 (Novus Biologicals, Littleton, CO, USA).Plasmid pcDNA3-HA-Wnt5a was a kind gift from Dr. M. Sen 45. Plasmid pUNO-GFP-hTLR4, pUNO1-hTLR02-DN and pUNO1-hTLR04-DN (Invivogen), human IL10 (hIL10) promoter plasmid was a kind gift from Professor L. Ziegler-Heitbrock, mouse IL10 (mIl10) promoter plasmid 41 (Addgene, Cambridge, MA, USA), AP-1-reporter kit (BPSBioscience, San Diego, CA, USA), TK-Renilla-promoter (Promega, Madison, WI, USA).Chloroquine (CQ) (InvivoGen, Toulouse, France) Dishevelled inhibitors NSC (Tocris Bio-Techne, Abington, UK) and Dvl (Merck, Darmstadt, Germany) .THP1- Blue™ NFκB cells and THP1-Dual™ KO-MyD (Invivogen).

Antibodies

Antibodies used

Commercial antibodies pAb-hTLR4 (pab-hstlr4), pAb-hTLR2 (pab-hstlr2), or a pAb-Control (pab-sctr) (Invivogen, Toulouse, France), or anti-HA-Alexa 594 1:2000 (Biolegend, San Diego, CA, USA). anti-Wnt5a 1:2000 (clone AF645) from R&D Systems (Minneapolis, MN, USA) and anti-His (H-3, sc-8036) from Santa Cruz Biotechnology Inc (Dallas, TX, USA) was used. In the western blots anti-pp38 (D-8), anti-p38 (C-20), anti-ERK (C-9), anti-IκBα (H-4) from Santa Cruz Biotechnology Inc all used at 1:200 (Dallas, TX, USA), anti-pERK1/2 (T202), anti-pAkt (D9E), anti-Akt (40D4), from Cell Signaling Technology all used at 1:1000 (Leiden, Netherlands) were used.

Validation

All antibodies were validated by proper controls.

Eukaryotic cell lines

Policy information about cell lines

Cell line source(s)

Human THP1 cells (ATCC® TIB-202TM, ATCC, LGC Standards, Middlesex, UK), and murine RAW264.7 macrophages (ATCC®-TIB-71TM). THP1- Blue™ NFκB cells and THP1-Dual™ KO-MyD (Invivogen).

Authentication

The cells were bought directly from commercial sources but not authenticated by us.

Mycoplasma contamination

The cell lines were tested for mycoplasma and were negative.

Commonly misidentified lines
(See ICLAC register)

The cell lines are NOT listed in the iclac register

Animals and other organisms

Policy information about studies involving animals; ARRIVE guidelines recommended for reporting animal research

Laboratory animals

The tibiae and femurs from WT (C57BL/6), MyD88-/-, and Tlr4-/- mice bred in the C57BL/6 background. male and female.Tlr4-/- mice (Tacon-IC, Hudson, NY) or MyD88-/- mice were bred in the C57Bl/6 background, and maintained at the Department of Microbiology, Immunology, and Glycobiology (MyD88-/-) or the Biomedical Center (Tlr4-/-) at Lund University. The study is in compliance with all relevant ethical regulations.

Wild animals

n/a

Field-collected samples

n/a

Human research participants

Policy information about studies involving human research participants

Population characteristics

Only healthy donors. Male and female. Permission for the study was obtained from the Regional Ethics Committee at Lund University (Dnr (registration number) 2012/689 and Dnr 2014/669) and the participating individuals provided a written informed consent before the study.

Recruitment

Blood donors at transfusion medicine. The participating individuals provided a written informed consent before the study.

Flow Cytometry

Plots

Confirm that:

☒ The axis labels state the marker and fluorochrome used (e.g. CD4-FITC).

☒ The axis scales are clearly visible. Include numbers along axes only for bottom left plot of group (a 'group' is an analysis of identical markers).

☒ All plots are contour plots with outliers or pseudocolor plots.

☒ A numerical value for number of cells or percentage (with statistics) is provided.

Methodology

Sample preparation	Monocytes were differentiated to M1 macrophages using rhGM-CSF for 5 d, and subsequently treated with rhIFN-γ and LPS (S. Typhimurium), rhHMGB1, or rhS100A9 for additional 2 d. During the experiment, rWnt5a was added on days 1 and 3. Cells were collected on day 7 using non-enzymatic cell dissociation solution (Sigma Aldrich).
Instrument	FACS verse
Software	FloJo
Cell population abundance	At least 100000 cells were seeded and all analyzed.
Gating strategy	See Suppl Fig 1.

☒ Tick this box to confirm that a figure exemplifying the gating strategy is provided in the Supplementary Information.
